# Health System Performance for Multimorbid Cardiometabolic Disease in India: A Population-Based Cross-Sectional Study

**DOI:** 10.5334/gh.1056

**Published:** 2022-01-31

**Authors:** Pascal Geldsetzer, Jan-Walter De Neve, Viswanathan Mohan, Dorairaj Prabhakaran, Ambuj Roy, Nikhil Tandon, Justine I. Davies, Sebastian Vollmer, Till Bärnighausen, Jonas Prenissl

**Affiliations:** 1 Division of Primary Care and Population Health, Department of Medicine, Stanford University, Stanford, CA, US; 2 Heidelberg Institute of Global Health, Medical Faculty and University Hospital, University of Heidelberg, Heidelberg, DE; 3 Madras Diabetes Research Foundation, ICMR Center for Advanced Research on Diabetes and Dr. Mohan’s Diabetes Specialities Centre, IDF Centre of Excellence in Diabetes Care, Chennai, Tamil Nadu, IN; 4 Public Health Foundation of India, New Delhi, Delhi NCR, IN; 5 Department of Cardiology, All India Institute of Medical Sciences, Ansari Nagar, New Delhi, IN; 6 Department of Endocrinology and Metabolism, All India Institute of Medical Sciences, Ansari Nagar, New Delhi, IN; 7 Institute for Applied Health Research, Birmingham University, Birmingham, UK; 8 MRC/Wits Rural Public Health and Health Transitions Research Unit (Agincourt), School of Public Health, Faculty of Health Sciences, University of the Witwatersrand, Johannesburg, ZA; 9 Centre for Global Surgery, Department of Global Health, Stellenbosch University, Stellenbosch, South Africa; 10 Department of Economics & Centre for Modern Indian Studies, University of Goettingen, Göttingen, DE; 11 Department of Global Health and Population, Harvard T.H. Chan School of Public Health, Boston, Massachusetts, US; 12 Africa Health Research Institute, Mtubatuba, KwaZulu-Natal, ZA

**Keywords:** health system performance, India, cardiometabolic diseases, multimorbidity

## Abstract

**Background:**

The prevalence of multimorbidity in low- and middle-income countries (LMICs) is thought to be rising rapidly. Research on the state of healthcare for multimorbidity in LMICs is needed to provide an impetus for integration of care across conditions, a baseline to monitor progress, and information for targeting of interventions to those most in need. Focusing on multimorbid cardiometabolic disease in India, this study thus aimed to determine 1) the proportion of adults with co-morbid diabetes and hypertension who successfully completed each step of the chronic disease care continuum from diagnosis to control for both conditions, and 2) how having additional cardiovascular disease (CVD) risk factors is associated with health system performance along the care continuum for diabetes, hypertension, and co-morbid diabetes and hypertension.

**Methods:**

Using a nationally representative household survey carried out in 2015 and 2016 among women aged 15–49 years and men aged 15–54 years, we created a ‘cascade of care’ for diabetes, hypertension, and co-morbid diabetes and hypertension by determining the proportion of those with the condition who had been diagnosed, were on treatment, and achieved control. We used Poisson regression with a robust error structure to estimate how having additional cardiovascular disease (CVD) risk factors (diabetes, hypertension, current smoking, and obesity) was associated with reaching each cascade step for diabetes, hypertension, and co-morbid diabetes and hypertension.

**Findings:**

Seven hundred thirty-four thousand seven hundred ninety-four adults were included in the analysis. Among individuals with co-morbid diabetes and hypertension, 28·8% (95% CI, 26·7%–31·0%), 16·1% (95% CI, 14·4%–17·9%), and 3·7% (95% CI, 2·8%–4·9%) – with these proportions varying between states by a factor of 4·8, 7·9, and 56·8 – were aware, treated, and achieved control of both conditions, respectively. Men, adults with lower household wealth, and those living in rural areas were less likely to reach each cascade step. Having additional CVD risk factors generally did not increase the probability of reaching each cascade step for diabetes, hypertension, and co-morbid diabetes and hypertension, except that having concurrent diabetes increased the probability of successfully transitioning through the hypertension care cascade.

**Interpretation:**

While varying widely between states and population groups, health system performance for co-morbid diabetes and hypertension is generally low in India, and there appears to be little integration of care across CVD risk factors.

**Funding:**

European Research Council.

## Introduction

Efforts to improve health in low- and middle-income countries (LMICs) have largely focused on single conditions (e.g., HIV or malaria) or technologies (e.g., vaccines) [1,2]. The rising burden of non-communicable diseases (NCDs) in LMICs offers a unique opportunity to guide policy attention and funding streams towards strengthening health systems in a horizontal fashion across multiple diseases, because many of these chronic conditions tend to co-occur and require long-term person-centered care [3]. Efforts to move towards health systems that can provide high-quality care across NCDs and tailor care to individuals’ comorbidities are crucial in resource-poor settings because 1) health systems in LMICs are often weak and fragmented [4], 2) strengthening primary care to improve management of NCDs will likely also have benefits for infectious disease care, such as through a higher ability to deal with new epidemics (as, for example, the experience with the 2014 West Africa Ebola outbreak has shown [5]), and 3) the time and, compared to local incomes, financial burden of accessing care tends to be higher in LMICs compared to high-income settings [6], meaning that many patients with multimorbidity can ill-afford to visit a specialty clinic for each of their conditions.

Research has an important role to play in guiding this transition from health services that provide care for single diseases to systems that effectively care for the multi-morbid patient in LMICs. Specifically, describing the state of healthcare for those with multiple chronic conditions can provide 1) an impetus for policy makers to move towards integration of care across diseases, 2) a baseline that allows for monitoring of progress over time, and 3) important information for the appropriate targeting of interventions to those most in need. This study aims to provide this evidence for cardiovascular disease (CVD) risk factors in India.

India faces a rapidly increasing burden of CVD [7]. Already the number one cause of death in the country [8], CVD is also the leading cause of disability-adjusted life years (DALYs) nationally [7,9]. Yet, research attention on CVD risk in India has often focused on the health system’s performance in managing CVD risk factors separately [10,11,12]. This study has a special focus on the detection and treatment of diabetes and hypertension because they are both major CVD risk factors [13,14,15], tend to co-occur [16], and are imminently treatable [17,18,19,20,21]. Furthermore, the additional CVD risk that both conditions confer appears to be at least partially preventable through effective glycemic and blood pressure (BP) control [22,23]. We have previously quantified the degree of co-occurrence of raised blood glucose and BP in India using population-based surveys conducted between 2012 and 2016, in which we found that 9% of individuals aged 65 years and older had both conditions concurrently [24]. To our knowledge, however, there has been no population-based analysis to date on the current state of healthcare in India for multiple concurrent CVD risk factors, including co-morbid diabetes and hypertension (a term we use in this manuscript to refer to having both conditions simultaneously).

Using a nationally representative sample of adults aged 15–49 years for women and 15–54 years for men, the aims of this study were threefold. First, we determined the proportion of adults with co-morbid diabetes and hypertension who successfully completed each step of the chronic disease care continuum from detection to successful treatment. Second, we examined how completion of these steps for co-morbid diabetes and hypertension varied between population groups and states within India. Third, because it is desirable for those with additional CVD risk factors – and thus a higher global CVD risk – to receive more intensive management of each CVD risk factor (e.g., more frequent screening for diabetes among those with obesity and more intensive control of hypertension among those with diabetes), we assessed how the proportion who successfully completed each step of the care continuum for diabetes or hypertension was associated with having additional CVD (diabetes, hypertension, current smoking, and obesity) risk factors.

## Methods

### Data source

Data used for this analysis was retrieved from the fourth National Family Health Survey (NFHS-4), which covered all districts in all states and Union Territories of India [25]. The survey was carried out between 2015 and 2016. It used a two-stage sampling process. Primary sampling units (PSUs; villages in rural and census enumeration blocks in urban areas) were selected with probability proportional to population size in rural areas and with equal probability in urban areas. In the second stage, twenty-two households were selected in each PSU using systematic random sampling, whereby the first household was selected randomly and then every *x*^th^ household was sampled. The data collection team administered a questionnaire to the household head and all non-pregnant women aged 15–49 years who had stayed in the household the night prior to the survey. These women were also eligible for height, weight, BP, and blood glucose measurements. Participants were not instructed to fast prior to the survey team’s visit. Because of its focus on maternal and child health, the NFHS-4 sampled men in only a random subsample of 15% of selected households. In these households, all men aged 15–54 years were eligible for a questionnaire and the same set of physical and biomarker measurements as women. We were, thus, unable to include older age groups in our analysis because these were not sampled in the NFHS-4. The response rate was 96·7% and 91·9% among women and men, respectively. More details on the survey methodology have been published elsewhere [26].

### Ascertaining hypertension and diabetes

BP was measured using the portable Omron HEM-8712 BP monitor three times on the left upper arm with at least five minutes between each BP measurement and five minutes of sitting prior to the first measurement. The mean of all three measurements was used in the analysis. If one measurement was missing (2·3% of participants), then the mean was calculated using the remaining two measurements. If two measurements were missing (1·4% of participants), the remaining measurement was used. Hypertension was defined as having a raised BP or having responded with ‘yes’ to either of the following two questions: ‘Were you told on two or more different occasions by a doctor or other health professional that you had hypertension or high blood pressure?’ and ‘To lower your blood pressure, are you now taking a prescribed medicine?’ [27] Raised BP was defined as mean systolic BP ≥140 mmHg or mean diastolic BP ≥90 mmHg [28].

Capillary blood glucose was measured using the Abbot Laboratories’ FreeStyle Optium H portable blood glucometer. The one-time capillary whole blood glucose measurement was converted into a plasma-equivalent glucose by multiplying with 1·11 [29]. Diabetes was then defined as having responded with ‘yes’ to the question ‘Do you currently have diabetes?’ or having a high plasma-equivalent glucose measurement (≥200 mg/dL [11·1 mmol/L] if reported not to have fasted, or ≥126 mg/dL [7·0 mmol/L] if reported to have fasted) [30]. Fasting was defined as having reported to not have eaten nor drunk anything besides plain water for at least eight hours. Participants were not instructed to fast, and thus only 1·3% were fasted at the time of the capillary blood glucose measurement.

### Construction of the care cascade

We computed the percentage of those who had both diabetes and hypertension who were 1) aware of having both conditions, 2) treated for both conditions, and 3) had achieved control for both conditions. We henceforth refer to this assessment as the ‘care cascade.’ Awareness of one’s diabetes and hypertension diagnosis was defined as having responded with ‘yes’ to the question ‘Do you currently have diabetes?’ and ‘Were you told on two or more different occasions by a doctor or other health professional that you had hypertension or high blood pressure?’ Because the wording of the question for diabetes (‘do you currently have diabetes?’) does not allow us to conclude which participants were formally diagnosed with diabetes, we chose to use the term ‘aware’ rather than ‘diagnosed’ in this manuscript. A participant was considered to be treated if the person had responded with ‘yes’ to the question ‘Have you sought treatment for this issue [diabetes]?’ (for diabetes) and ‘To lower your blood pressure, are you now taking a prescribed medicine?’ (for hypertension). Having achieved control was defined as being ‘treated’ plus having a plasma-equivalent blood glucose below 182 mg/dL (for diabetes), and a systolic BP <140 mmHg and diastolic BP <90 mmHg (for hypertension). There are no established criteria for assessing diabetes control with a random blood glucose measurement, because this commonly requires a glycated hemoglobin (HbA1c) measurement. The blood glucose cutoff used here is an approximation of an HbA1c measurement less than 8·0%, corresponding to the target of the American Diabetes Association [31]. HbA1c was not collected as part of the NFHS-4. We imposed that reaching a given cascade step was conditional on having reached the previous cascade step, which resulted in the exclusion of 0·0% and 2·2% of participants with the condition for diabetes and hypertension, respectively.

### Independent variables

We examined the association between the probability of reaching each care cascade step and the following socio-demographic variables: five-year age group, sex, rural-urban location, educational attainment, household wealth quintile, and marital status (currently married or not). We categorized educational attainment as ‘< primary school’ (which included those without any schooling and those who did not complete primary school), ‘Primary school completed,’ ‘Secondary school unfinished,’ and ‘Secondary school or above.’ The household wealth quintiles were based on a principal component analysis of questions on household ownership of 25 durable goods and seven key dwelling characteristics [32]. The continuous asset index and resulting quintiles were computed separately for rural and urban areas. Details on the computation of the household wealth quintiles are provided in eMethods1.

Furthermore, to determine whether those with additional CVD risk factors receive on average better management of diabetes and hypertension, we assessed the association between reaching each care cascade step (for diabetes, hypertension, and concurrent diabetes and hypertension) and having additional CVD risk factors, which were – apart from diabetes and hypertension – obesity and being a current smoker. Obesity was defined as having a Body Mass Index (BMI) ≥ 27·5 kg/m^2^ [33]. Currently smoking was defined as presently consuming any form of tobacco that is predominantly smoked (see eMethods2 for details). The NFHS-4 did not collect any information on blood lipids. We compared the association between the care cascade indicators and having additional CVD risk factors with that between the cascade indicators and two chronic conditions – anemia and asthma – which are not traditional CVD risk factors. This comparison was performed in an attempt to ascertain whether any positive associations between additional CVD risk factors and the care cascade for diabetes and hypertension are likely due to increased contact with the health system (because diabetes, hypertension, obesity, and smoking are all associated with conditions that would be expected to increase attendance at a healthcare facility) or instead due to targeted efforts at screening for, and treating each CVD risk factor more intensively among those who suffer from multiple CVD risk factors. Asthma was defined as having responded with ‘yes’ to the question ‘Do you currently have asthma?’ and anemia was defined as having an altitude- and smoking-adjusted hemoglobin <11·0 g/dl, corresponding to moderate or severe anemia as per the 2011 World Health Organization guidelines [34]. All participants underwent a hemoglobin measurement using the HemoCueHb 201+ photometer.

### Statistical analysis

We excluded pregnant women (54,153) from the analysis. All prevalence estimates used sampling weights to account for the survey design. These weights also accounted for the lower probability of sampling men. The care cascades described above were disaggregated by sex, rural-urban location, and the number of CVD risk factors that a participant had (diabetes, hypertension, obesity, and smoking). We also mapped the probability of reaching each cascade step by state and Union Territory, and then plotted these state-level probabilities against each state’s Gross Domestic Product (GDP) per capita to investigate whether health system performance for these conditions was related to a state’s wealth. Lastly, to examine how reaching each cascade step was associated with individual-level socio-demographic characteristics and co-morbidities, we used Poisson regressions with a robust error structure and – to filter out district-level effects – a binary indicator (‘fixed effect’) for each of India’s 640 districts [35]. We chose district-level fixed effects because the results can then be interpreted as depicting differences between population groups within districts, which could be used to inform district-level policy makers. Standard errors were adjusted for clustering at the PSU level [36]. This analysis was a complete case analysis. R software (version 3.3.2; R Foundation) was used for all statistical analyses.

## Results

### Sample characteristics

Seven hundred fifty-seven thousand six hundred fifty-five adults (647,451 women and 110,204 men) across 572,000 households participated in the survey. 3·0% (22,861/757,655) of all participants had a missing value for one of the care cascade-defining variables (BP, blood glucose, or questions on diagnosis and treatment of diabetes and hypertension) and were excluded from the analysis. Sample characteristics comparing included to excluded individuals can be found in eTable1. The final sample used for this analysis thus consisted of 734,794 participants (628,997 women and 105,797 men). An unweighted 2·8% of all participants had diabetes and 17·7% had hypertension (***Table 1***). One-third (33·6%) of the sample were aged 15 to 24 years, 79·4% did not finish secondary school, and 29·5% lived in urban areas. More women than men were obese whereas more men than women consumed tobacco (9·2% versus 7·1% and 2·1% versus 27·7%, respectively).

**Table 1:** Sample characteristics.^1^


CHARACTERISTIC	TOTAL	FEMALE	MALE

N	734794	628997	105797

Diabetes, n (%)	20300 (2·8)	16186 (2·6)	4114 (3·9)

Hypertension, n (%)	130324 (17·7)	108133 (17·2)	22191 (21·0)

Age Group, n (%), years			

15–19	131180 (17·9)	113255 (18·0)	17925 (16·9)

20–24	115584 (15·7)	100109 (15·9)	15475 (14·6)

25–29	112842 (15·4)	97751 (15·5)	15091 (14·3)

30–34	102252 (13·9)	88465 (14·1)	13787 (13·0)

35–39	98826 (13·4)	85618 (13·6)	13208 (12·5)

40–44	85079 (11·6)	73670 (11·7)	11409 (10·8)

45–49	80817 (11·0)	70129 (11·1)	10688 (10·1)

50–54	8214 (1·1)	–	8214 (7·8)

*Missing, n (%)*	0 (0·0)	0 (0·0)	0 (0·0)

Education, n (%)			

< Primary school	238788 (32·5)	217540 (34·6)	21248 (20·1)

Primary school completed	49127 (6·7)	42383 (6·7)	6744 (6·4)

Secondary school unfinished	295492 (40·2)	245899 (39·1)	49593 (46·9)

Secondary school or above	151387 (20·6)	123175 (19·6)	28212 (26·7)

*Missing, n (%)*	0 (0·0)	0 (0·0)	0 (0·0)

Household wealth quintile, n (%)			

Q1 (Poorest)	135426 (18·4)	117136 (18·6)	18290 (17·3)

Q2	145921 (19·9)	125363 (19·9)	20558 (19·4)

Q3	151445 (20·6)	129777 (20·6)	21668 (20·5)

Q4	149181 (20·3)	126996 (20·2)	22185 (21·0)

Q5 (Richest)	152821 (20·8)	129725 (20·6)	23096 (21·8)

*Missing, n (%)*	0 (0·0)	0 (0·0)	0 (0·0)

BMI, n (%)			

<18·5 kg/m^2^	159996 (21·8)	139780 (22·2)	20216 (19·1)

18·5–22·9 kg/m^2^	342101 (46·6)	291976 (46·4)	50125 (47·4)

23·0–24·9 kg/m^2^	96420 (13·1)	79872 (12·7)	16548 (15·6)

25·0–27·4 kg/m^2^	69622 (9·5)	58506 (9·3)	11116 (10·5)

27·5–29·9 kg/m^2^	35432 (4·8)	30735 (4·9)	4697 (4·4)

≥ 30·0 kg/m^2^	30189 (4·1)	27316 (4·3)	2873 (2·7)

*Missing, n (%)*	1034 (0·1)	812 (0·1)	222 (0·2)

Tobacco consumption, n (%)			

Current smoker	42487 (5·8)	13147 (2·1)	29340 (27·7)

*Missing, n (%)*	0 (0·0)	0 (0·0)	0 (0·0)

Currently married, n (%)	506850 (69·0)	440207 (70·0)	66643 (63·0)

*Missing, n (%)*	0 (0·0)	0 (0·0)	0 (0·0)

Urban area, n (%)	217024 (29·5)	183852 (29·2)	33172 (31·4)

*Missing, n (%)*	0 (0·0)	0 (0·0)	0 (0·0)


Abbreviations: n = number; Q = quintile. ^1^ The numbers and percentages in this table were not weighted using sampling weights.

### The cascade of care for co-morbid diabetes and hypertension

**Figure 1:**
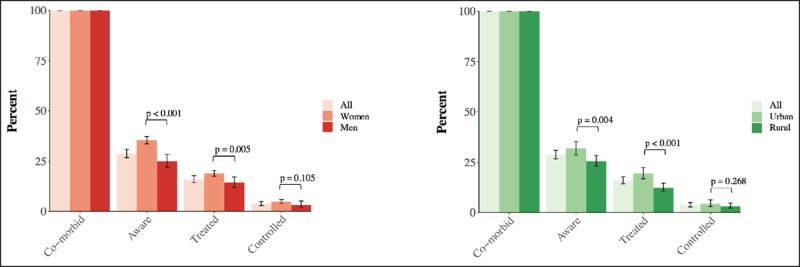
The cascade of care for co-morbid diabetes and hypertension in India, by sex and rural/urban location. ^1^ Each set of bars shows the percentage of those with co-morbid diabetes and hypertension who were aware of having both conditions, reported to be on treatment for both conditions, and achieved control of both conditions. ^2^ The p-values compare the proportion who reached each cascade step between men and women, and urban and rural areas. These were calculated using a Pearson’s Chi-Squared test with Rao and Scott adjustment. ^3^ The numbers plotted in this figure are shown in **eTable2–3**.

The prevalence of diabetes and hypertension was 3·7% (95% CI, 3·6%–3·9%) and 18·9% (95% CI, 18·6%–19·2%), respectively. 1·6% (95% CI, 1·5%–1·7%) had both diabetes and hypertension (i.e., ‘co-morbid’ diabetes and hypertension). Among individuals with co-morbid diabetes and hypertension, 28·8% (95% CI, 26·7%–31·0%) were aware of having both conditions, 16·1% (95% CI, 14·4%–17·9%) reported being treated for both, and 3·7% (95% CI, 2·8%–4·9%) achieved control of both their diabetes and hypertension (***Figure 1***). Women and those in urban areas had a higher probability of reaching each cascade step for their co-morbid diabetes and hypertension (but the difference was small and non-significant for achieving control).

### Geographic variation of the care cascade for co-morbid diabetes and hypertension

**Figure 2:**
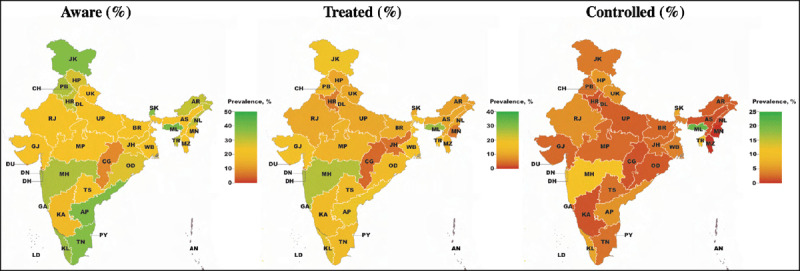
The cascade of care for co-morbid diabetes and hypertension in India by state.^1,2,3^ ^1^ Union Territories are included in the map but some are not visible due to their small size. ^2^ Point estimates and 95% confidence intervals for all states and Union Territories can be found in **eTable7–9**. ^3^ AN indicates Andaman and Nicobar Islands; AP, Andhra Pradesh; AR, Arunachal Pradesh; AS, Assam; BR, Bihar; CG, Chhattisgarh; CH, Chandigarh; DD, Daman and Diu; DL, Delhi; DN, Dadra and Nagar Haveli; GA, Goa; GJ, Gujarat; HR, Haryana; HP, Himachal Pradesh; JH, Jharkhand; JK, Jammu and Kashmir; KA, Karnataka; KL, Kerala; LD, Lakshadweep; MP, Madhya Pradesh; MH, Maharashtra; MN, Manipur; ML, Meghalaya; MZ, Mizoram; NL, Nagaland; OD, Odisha (Orissa); PB, Punjab; PY, Puducherry; RJ, Rajasthan; SK, Sikkim; TN, Tamil Nadu; TS, Telangana State; TR, Tripura; UP, Uttar Pradesh; UK, Uttarakhand (Uttaranchal); WB, West Bengal.

Among individuals with co-morbid diabetes and hypertension, being aware of both conditions ranged from 8·5% (95% CI, 5·1%–13·7%) in Chhattisgarh to 40·9% (40·9%, 95% CI: 25·9%–57·9%) in Meghalaya (***Figure 2***), being treated for both conditions from 3·6% (95% CI, 1·9%–6·7%) in Chhattisgarh to 28·6% in Meghalaya (95% CI, 15·9%–45·9%), and achieving control from 0·4% (95% CI, 0·1%–2·9%) in Manipur to 22·7% (95% CI, 10·8%–41·8%) in Meghalaya. There appeared to be no association between the proportion of those with co-morbid diabetes and hypertension who reached each cascade step and a state’s Gross Domestic Product (GDP) per capita (eFigure1 and eTable4–6).

### Individual-level variation of the care cascade for co-morbid diabetes and hypertension

The multivariable regression results in ***Table 2*** show that among those with co-morbid diabetes and hypertension 1) being female and living in an urban area was positively associated with reaching each care cascade step; 2) there was no clear association between age group and each cascade step; 3) while increasing household wealth quintile was positively associated with being aware and being treated for both conditions, higher educational attainment was only (positively and statistically significantly) associated with the awareness step. The results of univariable regressions (eTable10), regressions using sampling weights (eTable11), and when run separately by sex (eTable12–13) were similar.

**Table 2:** Individual-level predictors of reaching each cascade step among those with co-morbid diabetes and hypertension.^1,2^


	AWARE			TREATED			CONTROLLED	
		
	*RR (95% CI)*	*P*		*RR (95% CI)*	*P*		*RR (95% CI)*	*P*

Female	1·71 (1·52–1·92)	<0·001		1·85 (1·54–2·21)	<0·001		2·27 (1·55–3·32)	<0·001

Age group, y								

15–19	1·00 (Reference)			1·00 (Reference)			1·00 (Reference)	

20–24	1·15 (0·66–1·99)	0·631		0·50 (0·16–1·57)	0·235		0·40 (0·11–1·39)	0·148

25–29	1·08 (0·63–1·85)	0·778		0·76 (0·29–1·94)	0·56		0·38 (0·13–1·16)	0·088

30–34	0·95 (0·56–1·61)	0·843		0·62 (0·25–1·56)	0·314		0·14 (0·05–0·45)	0·001

35–40	1·08 (0·64–1·82)	0·778		0·94 (0·38–2·30)	0·894		0·24 (0·08–0·69)	0·008

40–44	1·14 (0·68–1·92)	0·617		1·23 (0·51–2·98)	0·649		0·28 (0·10–0·77)	0·014

45–49	1·38 (0·82–2·31)	0·224		1·70 (0·70–4·12)	0·239		0·37 (0·13–1·03)	0·058

50–54	1·71 (0·99–2·95)	0·055		1·99 (0·79–5·00)	0·145		0·42 (0·13–1·36)	0·148

Household wealth quintile								

Q1 (Poorest)	1·00 (Reference)			1·00 (Reference)			1·00 (Reference)	

Q2	1·13 (0·97–1·31)	0·115		1·39 (1·07–1·82)	0·015		0·94 (0·58–1·52)	0·804

Q3	1·22 (1·05–1·42)	0·008		1·43 (1·10–1·85)	0·008		0·84 (0·53–1·35)	0·482

Q4	1·35 (1·17–1·56)	<0·001		1·69 (1·30–2·19)	<0·001		1·12 (0·70–1·79)	0·628

Q5 (Richest)	1·43 (1·23–1·67)	<0·001		2·01 (1·54–2·63)	<0·001		1·23 (0·75–2·03)	0·414

Education								

< Primary school	1·00 (Reference)			1·00 (Reference)			1·00 (Reference)	

Primary school finished	1·09 (0·96–1·23)	0·175		1·12 (0·92–1·36)	0·248		1·17 (0·75–1·81)	0·482

Secondary school unfinished	1·13 (1·04–1·23)	0·006		1·05 (0·92–1·21)	0·441		0·92 (0·67–1·27)	0·625

Secondary school or above	1·19 (1·06–1·32)	0·002		1·13 (0·95–1·34)	0·159		1·25 (0·84–1·88)	0·276

Currently married	1·08 (0·96–1·20)	0·184		1·13 (0·95–1·35)	0·159		1·40 (0·94–2·08)	0·094

Urban	1·25 (1·15–1·35)	<0·001		1·63 (1·43–1·86)	<0·001		1·68 (1·25–2·26)	0·001


Abbreviations: RR = Risk Ratio; CI = Confidence Interval; Q = Quintile. ^1^ These regressions were run among those with co-morbid diabetes and hypertension, whereby ‘aware,’ ‘treated,’ and ‘controlled’ refers to being aware, treated, and controlled for both conditions. We used the same sample for all three regressions. That is, we did not restrict the sample for ‘treated’ to those who were aware of their co-morbid diabetes and hypertension. Neither did we restrict the sample for ‘controlled’ to those who were treated for their co-morbid diabetes and hypertension. ^2^ The regressions included all variables listed in the table and a binary indicator for each of 640 districts (district-level fixed effects) as independent variables. All standard errors were adjusted for clustering at the level of the primary sampling unit.

### Care cascades in relation to the number of CVD risk factors, and asthma and anemia

**Figure 3:**
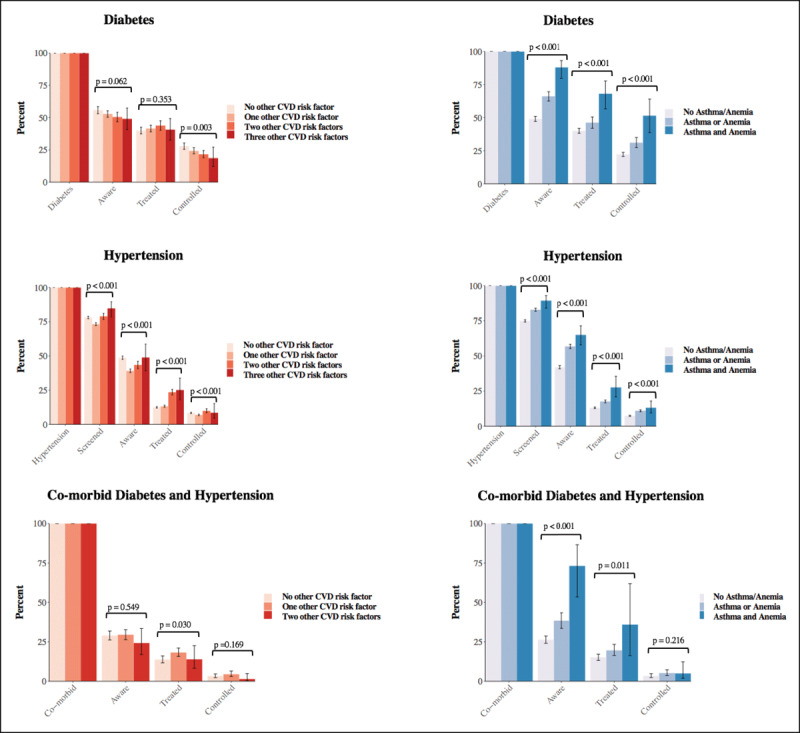
Care cascades in relation to the number of CVD risk factors as well as asthma and anemia.^1,2,3,4^ ^1^ CVD risk factors were diabetes, hypertension, current smoking, and obesity. ^2^ The p-values were for a one-way analysis of variance (with a Wald Chi-Squared test statistic) testing the null hypothesis that all bars in a set (i.e., separately for aware, treated, and controlled) were of the same height. ^3^ The numbers plotted in this figure are shown in **eTable14–16**. ^4^ Estimates separately for those with asthma but without anemia, with anemia but without asthma, and with both asthma and anemia are shown in **eFigure3**.

In general, the probability of reaching each cascade step for those with diabetes, hypertension, and co-morbid diabetes and hypertension did not increase with a rising number of CVD risk factors (***Figure 3***). In contrast, individuals with a non-CVD comorbidity – asthma and/or anemia – were more likely to reach each cascade step. Among current smokers, the number of CVD risk factors that a participant had was not associated with the probability of having received advice to quit smoking (eFigure2 and eTable17).

Multivariable regressions (***Table 3***) run separately for each care cascade step and separately for hypertension, diabetes, and co-morbid diabetes and hypertension, show that the magnitude of the associations between having multiple CVD risk factors and the probability of reaching each cascade step was relatively small – having RRs between 0·82 and 1·31 – with the exception of the comparatively strong positive association between having diabetes and reaching each step of the hypertension care cascade. Specifically, our regressions found that 1) diabetes was associated with better hypertension care cascade estimates, whereas hypertension was associated with a lower probability of reaching the ‘aware’ and ‘controlled’ steps of the diabetes cascade; 2) being a current smoker was not substantially associated with the probability of reaching a care cascade step for any of the cascade steps; and 3) being obese was generally associated with worse cascade indicators for the diabetes cascade but a higher probability of having reached the treatment step for hypertension, and co-morbid diabetes and hypertension (RR of 1·30 [95% CI, 1·26–1·34] and 1·32 [95% CI, 1·19–1·46], respectively). The results when running univariable regressions (eTable18), using sampling weights (eTable19), and running the regressions separately by sex (eTable20–21) were similar. Reporting to have asthma was associated with a higher probability of reaching each cascade step among those with diabetes, hypertension, and co-morbid diabetes and hypertension. Having anemia was associated with a higher probability of reaching each cascade step among participants with hypertension (except for having ever had one’s BP measured) and those with co-morbid diabetes and hypertension, but none of the cascade steps for those who had diabetes without having hypertension.

**Table 3:** Associations between having multiple cardiovascular disease risk factors (as well as asthma and anemia) and the probability of reaching each cascade step.^1,2^


	BP EVER MEASURED		AWARE OF HYPERTENSION		HYPERTENSION TREATED		HYPERTENSION CONTROLLED	

Has hypertension and …	*RR (95% CI)*	*P*	*RR (95% CI)*	*P*	*RR (95% CI)*	*P*	*RR (95% CI)*	*P*

diabetes	1·04 (1·04–1·05)	<0·001	1·12 (1·09–1·14)	<0·001	1·52 (1·47–1·58)	<0·001	1·33 (1·25–1·41)	<0·001

obesity	1·02 (1·01–1·03)	<0·001	1·01 (1·00–1·03)	0·063	1·30 (1·26–1·34)	<0·001	1·08 (1·04–1·13)	<0·001

smokes	0·98 (0·97–0·99)	<0·001	0·98 (0·96–1·00)	0·024	0·96 (0·92–1·00)	0·059	0·96 (0·91–1·02)	0·188

asthma	1·05 (1·04–1·07)	<0·001	1·14 (1·11–1·18)	<0·001	1·19 (1·12–1·27)	<0·001	1·15 (1·04–1·27)	0·004

anemia	1·02 (1·01–1·02)	<0·001	1·09 (1·08–1·10)	<0·001	1·12 (1·08–1·15)	<0·001	1·23 (1·18–1·28)	<0·001

			**AWARE OF DIABETES**		**DIABETES TREATED**		**DIABETES CONTROLLED**	

Has diabetes and …	–	–	*RR (95% CI)*	*P*	*RR (95% CI)*	*P*	*RR (95% CI)*	*P*

hypertension	–	–	0·96 (0·93–0·98)	0·002	1·00 (0·97–1·04)	0·817	0·83 (0·79–0·88)	<0·001

obesity	–	–	0·92 (0·89–0·94)	<0·001	0·94 (0·91–0·97)	0·001	0·82 (0·79–0·88)	<0·001

smokes	–	–	1·01 (0·97–1·05)	0·753	0·99 (0·94–1·04)	0·613	1·02 (0·95–1·10)	0·621

asthma	–	–	1·58 (1·53–1·63)	<0·001	1·58 (1·49–1·68)	<0·001	1·89 (1·76–2·03)	<0·001

anemia	–	–	1·02 (0·99–1·05)	0·245	1·01 (0·97–1·05)	0·715	0·99 (0·93–1·04)	0·642

			**AWARE OF BOTH**		**BOTH TREATED**		**BOTH CONTROLLED**	

Has diabetes, hypertension, and …	**–**	**–**	*RR (95% CI)*	*P*	*RR (95% CI)*	*P*	*RR (95% CI)*	*P*

obesity	–	–	1·03 (0·96–1·10)	0·393	1·31 (1·18–1·46)	<0·001	1·16 (0·91–1·48)	0·225

smokes	–	–	0·99 (0·88–1·09)	0·702	0·91 (0·77–1·08)	0·291	0·95 (0·64–1·39)	0·774

asthma	–	–	1·63 (1·47–1·81)	<0·001	1·37 (1·14–1·66)	0·001	1·93 (1·34–2·78)	<0·001

anemia	–	–	1·15 (1·07–1·25)	<0·001	1·25 (1·10–1·42)	0·001	1·29 (0·98–1·70)	0·070


Abbreviations: RR = Risk Ratio; CI = Confidence Interval; BP = Blood pressure. ^1^ These analyses depict – among each of the three samples (those with hypertension, those with diabetes, and those with co-morbid diabetes and hypertension) – the relative risk of reaching each care cascade step that is associated with having each additional CVD risk factor as well as asthma and anemia. ^2^ The regressions were run separately for each care cascade step and – in addition to the CVD risk factor, anemia or asthma shown in the table – contained age group, household wealth quintile, education, currently married, urban vs. rural location, sex, and a binary indicator for each of 640 districts (district-level fixed effects) as independent variables. Standard errors were adjusted for clustering at the level of the primary sampling unit.

## Discussion

This nationally representative study of individuals aged 15 to 54 years found that of those with co-morbid diabetes and hypertension, 28·8% were aware of both conditions, 16·1% were treated for both conditions, and 3·7% achieved control of both conditions. Within each of India’s 640 districts, men, adults with lower household wealth, and those living in rural areas were particularly likely to not have their need for care for co-morbid diabetes and hypertension met at each step of the care cascade. In addition, the cascade of care for co-morbid diabetes and hypertension varied greatly between states: by a factor of 4·8, 7·9, and 56·8 for the proportion being aware, treated, and controlled for both conditions, respectively. Surprisingly, wealthier states (as measured by GDP per capita) did not, on average, outperform poorer ones. Thus, the overall resource constraints of a state do not appear to be the main barrier to achieving at least some improvement in the care of those with co-morbid diabetes and hypertension. Our findings, therefore, suggest that important lessons can be learned from better-performing states in how screening, diagnosis, and treatment for co-morbid CVD care is furnished.

Having diabetes and hypertension, compared to having hypertension only, was associated with a higher probability of being aware, treated, and controlled for hypertension. This observation may stem from more frequent contact with the health system among those with diabetes (which, in turn, leads to more opportunities for clinicians to screen for, and treat, hypertension) or a higher attention to hypertension by clinicians among those with diabetes than those without diabetes. Apart from this observation, however, having additional CVD risk factors tended not to be associated with a greater probability of having received advice to quit smoking among smokers nor reaching each step of the care cascade among those with diabetes only, hypertension only, or co-morbid diabetes and hypertension. This finding is concerning because care for CVD risk factors should ideally be targeted at those with the highest global CVD risk. It thus appears that the Indian health system is currently failing to target their care efforts for control of CVD risk factors to those most in need. This result is surprising in light of the fact that one would expect those with a higher CVD risk – after adjusting for socio-demographic differences – to have more frequent contact with the health system, simply because, for instance, smoking and obesity are associated with a number of common chronic diseases [37,38,39,40]. Thus, in a health system in which healthcare workers provided some opportunistic CVD risk factor advice (e.g., to quit smoking) and screening, but did not target these efforts at those with a higher global CVD risk, we would still expect to have found a ‘better’ diabetes and hypertension care cascade as well as higher rates of having received advice to quit smoking among those with a greater number of CVD risk factors. Interestingly, having anemia or asthma – conditions that are not viewed as being traditional CVD risk factors but that may, on average, increase contact with the health system – was associated with better care cascade indicators for diabetes (asthma only), hypertension, and co-morbid diabetes and hypertension. Our findings therefore highlight the urgent need for the Indian health system to move towards a more person-centered primary healthcare approach – one that aims to prevent poor health outcomes by pro-actively screening and providing advice for conditions that a patient is likely to have and that compound the risk of adverse outcomes from conditions with which the patient has already been diagnosed. While such a shift in how healthcare is delivered will likely require broad reforms in the training and incentive structure for healthcare providers, it also presents an important opportunity to improve CVD care in the country with existing medications and technologies. In fact, India is currently undertaking major efforts to improve screening and care for diabetes and hypertension. Population-based screening for these conditions is being implemented as part of the National Program for Prevention and Control of Cancer, Diabetes, Cardiovascular Diseases and Stroke [41]. In addition, the provision of comprehensive primary care for diabetes and hypertension is envisioned under the Ayushman Bharat Health and Wellness Centre program through 150,000 health and wellness centers across the country [42].

While studies have examined the impact of multiple NCD morbidities on health service utilization and expenditure [43,44], this is – to our knowledge – the first population-based study from a LMIC to have examined the association between different CVD risk factors and the diabetes and hypertension care cascade. We can thus not comment on whether the patterns we have observed are unique to India. Nonetheless, there is evidence from the United States that multimorbidity is associated with better quality of care indicators for any one condition, including diabetes and hypertension [45,46], suggesting that our findings for India do not apply to the United States and possibly other high-income countries. A second unique contribution of this study is the in-depth examination of the current state of care in India for those with co-morbid diabetes and hypertension, and its variation between population groups. Whereas there have been some population-based studies on multimorbidity prevalence – including that of multiple CVD risk factors – in India [47,48,49], we are not aware of any other studies on the care cascade for those with co-morbid diabetes and hypertension.

This study has several limitations. First, this study only sampled women aged 15–49 years and men aged 15–54 years. Thus, our findings are only representative for this age group. This age restriction is also likely responsible for the lower prevalence of diabetes and hypertension observed in this sample compared to studies that included adults of all ages [50,51]. However, because India still has a comparatively young population structure, those aged 15–54 years account for the majority (75·2%) of all adults in India aged 15 years and older [52]. Nonetheless, restricting our analyses to these younger age groups misses those age groups with the highest CVD risk. Second, both hypertension and diabetes were not diagnosed according to the clinical gold standard. While a diagnosis of hypertension should ideally be based on BP measurements on two different occasions [53], the diagnosis for the purposes of this analysis was based on three measurements on a single occasion. Similarly, a diagnosis of diabetes should be based on a fasting plasma glucose measurement, an oral glucose tolerance test, or a glycated hemoglobin (HbA1c) assessment [54]. In clinical settings, a high (≥200 mg/dl) random blood glucose, which was the predominant measurement in this study, should only lead to a diagnosis of diabetes in the presence of symptoms. Third, glycemic control is typically assessed using a HbA1c measurement. The random blood glucose values that we had available may not be a good predictor of HbA1c. Fourth, the NFHS-4 did not collect any data on blood lipid levels. Fifth, men only constituted 14·4% of the total sample. Due to the large sample size, however, the absolute number of men (106,503) is still large enough to provide estimates with reasonable precision, and sampling weights were used to account for the underrepresentation of men. Sixth, the question to ascertain treatment for diabetes (‘Have you sought treatment for this issue [diabetes]?’) did not specifically ask about whether treatment was indeed received and whether the participant was still receiving treatment for diabetes at the time of the survey. Lastly, this study uses the cascade of care to assess health system performance. We, of course, recognize that demand-side factors also play an important role in transitioning through the care cascade. Thus, while we adjusted for individuals’ socio-demographic characteristics, differences in demand for care between individuals with single and multiple CVD risk factors that are not captured by people’s socio-demographic characteristics could be a confounder of the association (or lack thereof) between having additional CVD risk factors and reaching each step of the care cascade for diabetes, hypertension, and co-morbid diabetes and hypertension.

The co-occurrence of multiple CVD risk factors – and multimorbidity in general – is highly prevalent in high-income countries [3], and thought to be on the rise in LMICs like India [55,56,57]. To effectively deal with the rising prevalence of multiple risk factors and morbidities, health systems in LMICs must move away from episodic disease-specific care towards integrated person-centered care. Using a nationally representative population-based sample of individuals aged 15 to 54 years, this study shows that for those with co-morbid diabetes and hypertension, India’s health system is generally performing poorly. We also find that the health system currently does not appear to be effectively targeting screening and treatment efforts for CVD risk factors at those with the highest CVD risk. However, the cascade of care for co-morbid diabetes and hypertension varied widely among states – variation that appears to be independent of economic development – implying that important policy lessons could be learned from better-performing states. With the country accounting for over one sixth of the world’s population [52], whether or not global goals to control CVD risk factors and reduce premature mortality from CVD can be met will depend to a substantial degree on India’s ability to improve detection, treatment, and control of CVD risk factors, particularly among those with multiple CVD risk factors.

## Additional File

The additional file for this article can be found as follows:

10.5334/gh.1056.s1 Supplemental Appendix. The state of healthcare for adults with multiple cardiovascular disease risk factors in India: a population-based study of 740,000 adults.

## Data Accessibility Statement

The research published in this article is based on publicly available datasets, available at *https://dhsprogram.com/data/dataset/India_Standard-DHS_2015.cfm?flag=1*.

## Code Availability

The analysis code is available on the Harvard Dataverse (*https://doi.org/10.7910/DVN/MPEBLA*).
