# Supplementary material for: Health System Performance for Multimorbid Cardiometabolic Disease in India: A Population-Based Cross-Sectional Study
Source: Glob Heart. 2022 Jan 31;17(1):7. doi: 10.5334/gh.1056 (PMC8815445; doi:10.5334/gh.1056)
Supplement: Supplemental Appendix. — The state of healthcare for adults with multiple cardiovascular disease risk factors in India: a population-based study of 740,000 adults. [file gh-17-1-1056-s1.pdf]

Supplemental Appendix

The state of healthcare for adults with multiple cardiovascular disease risk factors in India: a population-based study of 740,000 adults

eMethods1. Computation of the household wealth index 3

eMethods2. Defining the variable 'current smoking ' 5

eTable1. Sample characteristics of excluded individuals[1] 6

eTable2. Combined cascade estimates by sex.. 7

eTable3. Combined cascade estimates by rural/urban location 8

eTable4. Combined care cascade: 'Aware' estimates by state and GDP per capita 9

eTable5. Combined care cascade: 'Treated' estimates by state and GDP per capita 10

eTable6. Combined care cascade: 'Controlled' estimates by state and GDP per capita 11

eTable7. Combined care cascade: 'Aware' estimates by state.. 12

eTable8. Combined care cascade: 'Treated' estimates by state 13

eTable9. Combined care cascade: 'Controlled' estimates by state.. 14

eTable10. Univariable regressions: Individual-level correlates of reaching each cascade step among those with co-morbid diabetes and hypertension[1,2] 15

eTable11. Individual-level correlates of reaching each cascade step among those with co-morbid diabetes and hypertension, using sampling weights[1,2]... 18

eTable12. Individual-level correlates of reaching each cascade step among those with co-morbid diabetes and hypertension, women only[1,2].. 21

eTable13. Individual-level correlates of reaching each cascade step among those with co-morbid diabetes and hypertension, men only[1,2]... 24

eTable14. Diabetes cascade estimates with comorbidities.. 27

eTable15. Hypertension cascade estimates with comorbidities... 28

eTable16. Combined cascade estimates with comorbidities.. 29

eTable17. 'Treated' for tobacco consumption with comorbidities 30

eTable 18. Univariable regressions: The association of having multiple cardiovascular disease risk factors with the probability of reaching each cascade step[1] 31

eTable 19. The association of having multiple cardiovascular disease risk factors with the probability of reaching each cascade step using sampling weights[1].................................... 33

eTable 20. The association of having multiple cardiovascular disease risk factors with the probability of reaching each cascade step, men only[1]... 35

eTable 21. The association of having multiple cardiovascular disease risk factors with the probability of reaching each cascade step, women only[1] 37

eFigure 1. Combined care cascade: states' gross domestic product (GDP) per capita and each care cascade step[1,2,3,4,5,6,7] 39

eFigure 2. 'Treated' for smoking tobacco with additional CVD-risk factors or non-CVD morbidites[1,2] 41

eMethods1. Computation of the household wealth index

Multiple dwelling characteristics were used to create the household wealth index:

- Main roof material
- Main wall material
- Main material of floor
- Type of cooking fuel
- Type of toilet facility
- Source of drinking water

Furthermore, durable goods ('assets') ownership of the following items was analyzed:

- Mattress
- Pressure cooker
- Chair
- Cot or bed
- Table
- Electric fan
- Radio or transistor
- Black and white television
- Colour television
- Sewing machine
- Mobile telephone
- Telephone (non-mobile)
- Internet
- Computer
- Refrigerator
- Air conditioner/cooler
- Washing machine

- Watch or clock
- Bicycle
- Motorcycle or Scooter
- Animal-drawn cart
- Car
- Water pump
- Thresher
- Tractor

First, all items listed above were encoded as a binary indicator. Second, a Principal Component Analysis (PCA) was performed separately for rural and urban areas and the first (unrotated) component was selected. The resulting standardized score has a mean of zero, a standard deviation of one, and wealthier households receive higher scores. To improve interpretability, the variable was then divided into quintiles. A description of the exact calculation process used for the DHS surveys has been published.(1, 2)

eMethods2. Defining the variable 'current smoking '

Multiple questionnaire items were cumulated to create the variable current smoking. All items used were self-reported. Current smoking was defined as having selected 'Cigar', 'Pipe' or 'Hookah' as answer for the question "In what other form do you currently smoke or use tobacco?" or having responded with 'yes' to the question "Do you currently smoke cigarettes?" or "Do you currently smoke bidis?".

eTable1. Sample characteristics of excluded individuals[1]

	Total	Female	Male
n	22861	18454	4407
Diabetes, n (%)	146 (0.6)	122 (0.7)	24 (0.5)
Hypertension, n (%)	1069 (4.7)	919 (5.0)	150 (3.4)
Age Group, n (%), years			
15 - 19	4789 (20.9)	4004 (21.7)	785 (17.8)
20 - 24	3747 (16.4)	3040 (16.5)	707 (16.0)
25 - 29	3489 (15.3)	2782 (15.1)	707 (16.0)
30 - 34	2951 (12.9)	2389 (12.9)	562 (12.8)
35 - 39	2743 (12.0)	2258 (12.2)	485 (11.0)
40 - 44	2440 (10.7)	2001 (10.8)	439 (10.0)
45 - 49	2380 (10.4)	1980 (10.7)	400 (9.1)
50 - 54	322 (1.4)	0 (0.0)	322 (7.3)
Education, n (%)			
Primary school unfinished	6329 (27.7)	5536 (30.0)	793 (18.0)
Primary school finished	1255 (5.5)	1021 (5.5)	234 (5.3)
Secondary school unfinished	9199 (40.2)	7168 (38.8)	2031 (46.1)
Secondary school finished or above	6078 (26.6)	4729 (25.6)	1349 (30.6)
Household wealth quintile, n (%)			
Q1 (Poorest)	3897 (17.0)	3174 (17.2)	723 (16.4)
Q2	4174 (18.3)	3352 (18.2)	822 (18.7)
Q3	4505 (19.7)	3652 (19.8)	853 (19.4)
Q4	4687 (20.5)	3725 (20.2)	962 (21.8)
Q5 (Richest)	5598 (24.5)	4551 (24.7)	1047 (23.8)
BMI, n (%)			
<18.5kg/m^2	4380 (19.2)	3737 (20.3)	643 (14.6)
18.5 - 22.9 kg/m^2	2119 (9.3)	1889 (10.2)	230 (5.2)
23.0 - 24.9 kg/m^2	1182 (5.2)	977 (5.3)	205 (4.7)
25.0 - 27.4 kg/m^2	878 (3.8)	744 (4.0)	134 (3.0)
27.5 - 29.9 kg/m^2	504 (2.2)	437 (2.4)	67 (1.5)
≥ 30.0 kg/m^2	421 (1.8)	380 (2.1)	41 (0.9)
Missing	13377 (58.5)	10290 (55.8)	3087 (70.0)
Tobacco consumption, n (%)			
Current smoker	1391 (6.1)	260 (1.4)	1131 (25.7)
Uses smokeless tobacco	2451 (10.7)	1189 (6.4)	1262 (28.6)
Currently married, n (%)	13974 (61.1)	11483 (62.2)	2491 (56.5)
Urban area, n (%)	9530 (41.7)	7630 (41.3)	1900 (43.1)

Abbreviations: n=number; Q=quintile.

[1] The numbers and percentages in this table were not weighted using sampling weights.

eTable2. Combined cascade estimates by sex

Care indicator	Sex	Estimate (in %)	Low CI (in %)	High CI (in %)
Aware	All	28.82	26.74	30.98
Treated	All	16.07	14.40	17.90
Controlled	All	3.72	2.83	4.89
Aware	Women	35.48	33.67	37.32
Treated	Women	18.99	17.57	20.49
Controlled	Women	4.78	3.99	5.72
Aware	Men	25.13	22.13	28.40
Treated	Men	14.46	12.06	17.24
Controlled	Men	3.14	1.93	5.07

eTable3. Combined cascade estimates by rural/urban location

Care indicator	Location	Estimate (in %)	Low CI (in %)	High CI (in %)
Aware	All	28.82	26.74	30.98
Treated	All	16.07	14.40	17.90
Controlled	All	3.72	2.83	4.89
Aware	Urban	31.89	28.69	35.26
Treated	Urban	19.57	16.97	22.45
Controlled	Urban	4.28	2.95	6.19
Aware	Rural	25.66	23.11	28.40
Treated	Rural	12.48	10.55	14.72
Controlled	Rural	3.15	2.11	4.68

eTable4. Combined care cascade: 'Aware' estimates by state and GDP per capita

State	Estimate (in %)	Low CI (in %)	Upper CI (in %)	Zone	Label	GDP per capita (inter. Dollar)
Andhra Pradesh	38.44	29.43	48.31	South	AP	5616.677959
Arunachal Pradesh	29.81	18.91	43.62	Northeast	AR	5849.635825
Assam	21.93	16.07	29.19	Northeast	AS	3053.653753
Bihar	24.22	18.11	31.60	East	BR	1972.806858
Chhattisgarh	8.50	5.14	13.73	Central	CT	4343.709448
Goa	22.08	10.27	41.22	West	GA	20033.76694
Gujarat	22.81	15.62	32.06	West	GJ	7570.097496
Haryana	29.38	19.61	41.51	North	HR	9167.568268
Himachal Pradesh	27.63	17.62	40.52	North	HP	7189.290719
Jammu and Kashmir	36.43	26.86	47.20	North	JK	4172.65962
Jharkhand	24.62	17.18	33.97	East	JH	3129.812781
Karnataka	17.40	11.33	25.77	South	KA	6011.598363
Kerala	35.33	25.46	46.63	South	KL	7088.907536
Madhya Pradesh	23.36	18.11	29.57	Central	MP	3577.032479
Maharashtra	32.71	23.53	43.45	West	MH	8030.600448
Manipur	21.71	12.44	35.12	Northeast	MN	2997.351466
Meghalaya	40.91	25.85	57.89	Northeast	ML	4415.492083
Mizoram	23.73	14.01	37.27	Northeast	MZ	5608.190718
Nagaland	30.74	19.27	45.21	Northeast	NL	5360.899074
Odisha	26.66	20.09	34.45	East	OD	3886.408533
Punjab	33.25	24.85	42.86	North	PB	6840.090457
Rajasthan	24.46	16.99	33.87	North	RJ	4512.420172
Sikkim	37.48	21.70	56.47	Northeast	SK	12113.65458
Tamil Nadu	37.77	31.79	44.15	South	TN	7075.557494
Telangana	23.19	15.00	34.07	South	TS	6651.843008
Tripura	28.93	15.89	46.73	Northeast	TR	4360.81546
Uttar Pradesh	23.81	19.47	28.78	Central	UP	2580.244624
Uttarakhand	23.82	15.63	34.53	Central	UK	7281.317689
West Bengal	24.41	17.07	33.63	East	WB	4625.86283

eTable5. Combined care cascade: 'Treated' estimates by state and GDP per capita

State	Estimate (in %)	Low CI (in %)	Upper CI (in %)	Zone	Label	GDP per capita (inter. Dollar)
Andhra Pradesh	21.41	14.60	30.26	South	AP	5616.677959
Arunachal Pradesh	9.07	3.56	21.23	Northeast	AR	5849.635825
Assam	10.99	7.05	16.73	Northeast	AS	3053.653753
Bihar	11.68	7.21	18.39	East	BR	1972.806858
Chhattisgarh	3.55	1.86	6.67	Central	CT	4343.709448
Goa	22.17	10.35	41.28	West	GA	20033.76694
Gujarat	17.77	11.30	26.84	West	GJ	7570.097496
Haryana	4.71	2.58	8.45	North	HR	9167.568268
Himachal Pradesh	11.34	5.27	22.72	North	HP	7189.290719
Jammu and Kashmir	19.51	12.31	29.51	North	JK	4172.65962
Jharkhand	5.30	2.95	9.33	East	JH	3129.812781
Karnataka	15.48	9.39	24.44	South	KA	6011.598363
Kerala	20.68	13.39	30.52	South	KL	7088.907536
Madhya Pradesh	13.42	9.27	19.05	Central	MP	3577.032479
Maharashtra	26.64	17.79	37.85	West	MH	8030.600448
Manipur	5.42	2.30	12.23	Northeast	MN	2997.351466
Meghalaya	28.60	15.90	45.92	Northeast	ML	4415.492083
Mizoram	12.62	6.38	23.44	Northeast	MZ	5608.190718
Nagaland	10.29	4.02	23.88	Northeast	NL	5360.899074
Odisha	14.57	10.20	20.38	East	OD	3886.408533
Punjab	13.89	9.03	20.78	North	PB	6840.090457
Rajasthan	11.83	6.96	19.39	North	RJ	4512.420172
Sikkim	11.83	6.71	20.00	Northeast	SK	12113.65458
Tamil Nadu	15.22	11.64	19.65	South	TN	7075.557494
Telangana	14.41	8.36	23.71	South	TS	6651.843008
Tripura	18.00	7.44	37.50	Northeast	TR	4360.81546
Uttar Pradesh	10.33	7.71	13.70	Central	UP	2580.244624
Uttarakhand	11.22	6.44	18.82	Central	UK	7281.317689
West Bengal	19.03	12.40	28.08	East	WB	4625.86283

eTable6. Combined care cascade: 'Controlled' estimates by state and GDP per capita

State	Estimate (in %)	Low CI (in %)	Upper CI (in %)	Zone	Label	GDP per capita (inter. Dollar)
Andhra Pradesh	4.78	2.04	10.77	South	AP	5616.677959
Arunachal Pradesh	1.58	0.38	6.39	Northeast	AR	5849.635825
Assam	1.42	0.57	3.51	Northeast	AS	3053.653753
Bihar	2.60	1.50	4.48	East	BR	1972.806858
Chhattisgarh	1.41	0.44	4.38	Central	CT	4343.709448
Goa	2.21	0.88	5.43	West	GA	20033.76694
Gujarat	2.52	0.92	6.70	West	GJ	7570.097496
Haryana	1.41	0.52	3.75	North	HR	9167.568268
Himachal Pradesh	5.31	1.16	21.18	North	HP	7189.290719
Jammu and Kashmir	3.51	1.92	6.33	North	JK	4172.65962
Jharkhand	2.79	1.16	6.55	East	JH	3129.812781
Karnataka	1.54	0.54	4.31	South	KA	6011.598363
Kerala	5.83	2.69	12.18	South	KL	7088.907536
Madhya Pradesh	2.47	1.02	5.89	Central	MP	3577.032479
Maharashtra	11.83	5.28	24.41	West	MH	8030.600448
Manipur	0.40	0.05	2.85	Northeast	MN	2997.351466
Meghalaya	22.71	10.75	41.75	Northeast	ML	4415.492083
Mizoram	0.95	0.35	2.58	Northeast	MZ	5608.190718
Nagaland	1.06	0.14	7.37	Northeast	NL	5360.899074
Odisha	1.95	1.12	3.36	East	OD	3886.408533
Punjab	2.75	0.79	9.19	North	PB	6840.090457
Rajasthan	4.67	1.56	13.10	North	RJ	4512.420172
Sikkim	0.00	0.00	0.00	Northeast	SK	12113.65458
Tamil Nadu	3.21	1.83	5.56	South	TN	7075.557494
Telangana	3.00	1.15	7.58	South	TS	6651.843008
Tripura	10.34	2.85	31.14	Northeast	TR	4360.81546
Uttar Pradesh	1.62	0.83	3.14	Central	UP	2580.244624
Uttarakhand	4.71	1.52	13.68	Central	UK	7281.317689
West Bengal	3.38	1.16	9.44	East	WB	4625.86283

eTable7. Combined care cascade: 'Aware' estimates by state

State	Estimate (in %)	Low CI (in %)	Upper CI (in %)
Andaman and Nicobar Islands	29.92	14.18	52.46
Andhra Pradesh	38.44	29.43	48.31
Arunachal Pradesh	29.81	18.91	43.62
Assam	21.93	16.07	29.19
Bihar	24.22	18.11	31.60
Chandigarh	31.45	7.99	70.80
Chhattisgarh	8.50	5.14	13.73
Dadra and Nagar Haveli	8.32	1.55	34.39
Daman and Diu	8.10	1.39	35.52
Delhi	34.55	18.86	54.53
Goa	22.08	10.27	41.22
Gujarat	22.81	15.62	32.06
Haryana	29.38	19.61	41.51
Himachal Pradesh	27.63	17.62	40.52
Jammu and Kashmir	36.43	26.86	47.20
Jharkhand	24.62	17.18	33.97
Karnataka	17.40	11.33	25.77
Kerala	35.33	25.46	46.63
Lakshadweep	23.80	7.63	54.15
Madhya Pradesh	23.36	18.11	29.57
Maharashtra	32.71	23.53	43.45
Manipur	21.71	12.44	35.12
Meghalaya	40.91	25.85	57.89
Mizoram	23.73	14.01	37.27
Nagaland	30.74	19.27	45.21
Odisha	26.66	20.09	34.45
Puducherry	41.37	20.39	66.03
Punjab	33.25	24.85	42.86
Rajasthan	24.46	16.99	33.87
Sikkim	37.48	21.70	56.47
Tamil Nadu	37.77	31.79	44.15
Telangana	23.19	15.00	34.07
Tripura	28.93	15.89	46.73
Uttar Pradesh	23.81	19.47	28.78
Uttarakhand	23.82	15.63	34.53
West Bengal	24.41	17.07	33.63

eTable8. Combined care cascade: 'Treated' estimates by state

State	Estimate (in %)	Low CI (in %)	Upper CI (in %)
Andaman and Nicobar Islands	26.60	12.35	48.26
Andhra Pradesh	21.41	14.60	30.26
Arunachal Pradesh	9.07	3.56	21.23
Assam	10.99	7.05	16.73
Bihar	11.68	7.21	18.39
Chandigarh	17.50	3.92	52.49
Chhattisgarh	3.55	1.86	6.67
Dadra and Nagar Haveli	5.42	0.78	29.58
Daman and Diu	8.10	1.39	35.52
Delhi	19.99	8.61	39.85
Goa	22.17	10.35	41.28
Gujarat	17.77	11.30	26.84
Haryana	4.71	2.58	8.45
Himachal Pradesh	11.34	5.27	22.72
Jammu and Kashmir	19.51	12.31	29.51
Jharkhand	5.30	2.95	9.33
Karnataka	15.48	9.39	24.44
Kerala	20.68	13.39	30.52
Lakshadweep	15.33	4.66	40.17
Madhya Pradesh	13.42	9.27	19.05
Maharashtra	26.64	17.79	37.85
Manipur	5.42	2.30	12.23
Meghalaya	28.60	15.90	45.92
Mizoram	12.62	6.38	23.44
Nagaland	10.29	4.02	23.88
Odisha	14.57	10.20	20.38
Puducherry	30.87	12.86	57.46
Punjab	13.89	9.03	20.78
Rajasthan	11.83	6.96	19.39
Sikkim	11.83	6.71	20.00
Tamil Nadu	15.22	11.64	19.65
Telangana	14.41	8.36	23.71
Tripura	18.00	7.44	37.50
Uttar Pradesh	10.33	7.71	13.70
Uttarakhand	11.22	6.44	18.82
West Bengal	19.03	12.40	28.08

eTable9. Combined care cascade: 'Controlled' estimates by state

State	Estimate (in %)	Low CI (in %)	Upper CI (in %)
Andaman and Nicobar Islands	1.31	0.17	9.45
Andhra Pradesh	4.78	2.04	10.77
Arunachal Pradesh	1.58	0.38	6.39
Assam	1.42	0.57	3.51
Bihar	2.60	1.50	4.48
Chandigarh	0.00	0.00	0.00
Chhattisgarh	1.41	0.44	4.38
Dadra and Nagar Haveli	0.00	0.00	0.00
Daman and Diu	0.00	0.00	0.00
Delhi	1.07	0.30	3.77
Goa	2.21	0.88	5.43
Gujarat	2.52	0.92	6.70
Haryana	1.41	0.52	3.75
Himachal Pradesh	5.31	1.16	21.18
Jammu and Kashmir	3.51	1.92	6.33
Jharkhand	2.79	1.16	6.55
Karnataka	1.54	0.54	4.31
Kerala	5.83	2.69	12.18
Lakshadweep	1.15	0.13	9.47
Madhya Pradesh	2.47	1.02	5.89
Maharashtra	11.83	5.28	24.41
Manipur	0.40	0.05	2.85
Meghalaya	22.71	10.75	41.75
Mizoram	0.95	0.35	2.58
Nagaland	1.06	0.14	7.37
Odisha	1.95	1.12	3.36
Puducherry	5.60	1.83	15.89
Punjab	2.75	0.79	9.19
Rajasthan	4.67	1.56	13.10
Sikkim	0.00	0.00	0.00
Tamil Nadu	3.21	1.83	5.56
Telangana	3.00	1.15	7.58
Tripura	10.34	2.85	31.14
Uttar Pradesh	1.62	0.83	3.14
Uttarakhand	4.71	1.52	13.68
West Bengal	3.38	1.16	9.44

eTable10. Univariable regressions: Individual-level correlates of reaching each cascade step among those with co-morbid diabetes and hypertension[1,2]

Aware

	RR	95% CI (low)	95% CI (high)	P Value
Age group, y				
15-19	1(Reference)			
20-24	1.28	0.73	2.24	0.390
25-29	1.18	0.69	2.03	0.547
30-34	1.06	0.63	1.80	0.825
35-40	1.20	0.71	2.03	0.485
40-44	1.29	0.77	2.17	0.330
45-49	1.56	0.93	2.61	0.093
50-54	1.25	0.73	2.13	0.416
Education				
Primary school unfinished	1(Reference)			
Primary school finished	1.10	0.97	1.24	0.144
Secondary school unfinished	1.12	1.03	1.21	0.006
Secondary school or above	1.17	1.06	1.28	0.001
Household wealth quintile				
Q1 (Poorest)	1(Reference)			
Q2	1.12	0.96	1.30	0.150
Q3	1.23	1.06	1.42	0.006
Q4	1.36	1.18	1.56	< 0.001
Q5 (Richest)	1.42	1.23	1.63	< 0.001
Currently married	1.07	0.96	1.19	0.221
Female	1.48	1.34	1.63	< 0.001
Urban	1.22	1.13	1.31	< 0.001

Treated

	RR	95% CI (low)	95% CI (high)	P Value
Age group, y				
15-19	1(Reference)			
20-24	1.34	0.39	4.66	0.644
25-29	3.56	1.23	10.25	0.019
30-34	4.12	1.47	11.54	0.007
35-40	7.65	2.80	20.95	< 0.001
40-44	11.69	4.30	31.80	< 0.001
45-49	18.12	6.68	49.11	< 0.001
50-54	14.17	5.12	39.20	< 0.001
Education				
Primary school unfinished	1(Reference)			
Primary school finished	1.21	0.99	1.48	0.058
Secondary school unfinished	1.06	0.93	1.20	0.398
Secondary school or above	1.10	0.94	1.29	0.217
Household wealth quintile				
Q1 (Poorest)	1(Reference)			
Q2	1.62	1.24	2.13	< 0.001
Q3	1.70	1.30	2.22	< 0.001
Q4	2.01	1.56	2.60	< 0.001
Q5 (Richest)	2.49	1.93	3.21	< 0.001
Currently married	1.63	1.36	1.95	< 0.001
Female	1.42	1.23	1.65	< 0.001
Urban	1.82	1.61	2.06	< 0.001

Controlled

	RR	95% CI (low)	95% CI (high)	P Value
Age group, y				
15-19	1(Reference)			
20-24	1.16	0.32	4.25	0.819
25-29	2.14	0.69	6.62	0.187
30-34	1.14	0.36	3.63	0.821
35-40	2.27	0.78	6.55	0.131
40-44	3.24	1.14	9.19	0.027
45-49	4.78	1.70	13.41	0.003
50-54	3.31	1.08	10.18	0.036
Education				
Primary school unfinished	1(Reference)			
Primary school finished	1.25	0.82	1.92	0.305
Secondary school unfinished	0.93	0.71	1.21	0.574
Secondary school or above	1.16	0.84	1.58	0.370
Household wealth quintile				
Q1 (Poorest)	1(Reference)			
Q2	1.05	0.67	1.67	0.824
Q3	1.00	0.63	1.60	0.984
Q4	1.27	0.82	1.96	0.287
Q5 (Richest)	1.41	0.91	2.19	0.123
Currently married	1.70	1.17	2.46	0.005
Female	1.69	1.25	2.29	< 0.001
Urban	1.91	1.48	2.47	< 0.001

Abbreviations: RR = Relative Risk; CI = Confidence Interval; Q = Quintile

[1] These regressions were run among those with co-morbid diabetes and hypertension, whereby aware, treated, and controlled refers to being aware, treated, and controlled for both conditions.

[2] The regressions included all variables listed in the table and an additional binary indicator for each of 640 districts (district-level fixed effects) as independent variables. All standard errors were adjusted for clustering at the level of a primary sampling unit.

eTable11. Individual-level correlates of reaching each cascade step among those with co-morbid diabetes and hypertension, using sampling weights[1,2]

Aware

	RR (95% CI)	P Value
Age group, y		
15-19	1(Reference)	
20-24	0.60 (0.38-0.95)	0.03
25-29	0.75 (0.51-1.12)	0.16
30-34	0.55 (0.37-0.82)	0.003
35-40	0.73 (0.50-1.07)	0.107
40-44	0.80 (0.55-1.16)	0.24
45-49	1.04 (0.71-1.51)	0.852
50-54	1.33 (0.91-1.95)	0.143
Education		
Primary school unfinished	1(Reference)	
Primary school finished	0.96 (0.85-1.07)	0.453
Secondary school unfinished	1.10 (1.02-1.18)	0.016
Secondary school or above	1.02 (0.93-1.12)	0.612
Household wealth quintile		
Q1 (Poorest)	1(Reference)	
Q2	1.20 (1.06-1.36)	0.004
Q3	1.31 (1.16-1.48)	<0.001
Q4	1.51 (1.34-1.71)	<0.001
Q5 (Richest)	1.83 (1.61-2.08)	<0.001
Currently married	1.02 (0.92-1.14)	0.698
Female	1.70 (1.59-1.81)	<0.001
Urban	1.43 (1.33-1.53)	<0.001

Treated

	RR (95% CI)	P Value
Age group, y		
15-19	1(Reference)	
20-24	0.53 (0.15-1.94)	0.34
25-29	0.73 (0.24-2.17)	0.566
30-34	0.94 (0.32-2.74)	0.906
35-40	1.19 (0.41-3.43)	0.752
40-44	1.90 (0.66-5.47)	0.231
45-49	2.71 (0.94-7.76)	0.064
50-54	3.72 (1.29-10.73)	0.015
Education		
Primary school unfinished	1(Reference)	
Primary school finished	1.04 (0.89-1.22)	0.611
Secondary school unfinished	1.09 (0.99-1.22)	0.093
Secondary school or above	1.02 (0.89-1.15)	0.813
Household wealth quintile		
Q1 (Poorest)	1(Reference)	
Q2	1.46 (1.21-1.76)	<0.001
Q3	1.56 (1.29-1.88)	<0.001
Q4	1.92 (1.60-2.32)	<0.001
Q5 (Richest)	2.63 (2.17-3.19)	<0.001
Currently married	1.22 (1.05-1.42)	0.009
Female	1.70 (1.55-1.85)	<0.001
Urban	1.85 (1.68-2.04)	<0.001

Controlled

	RR (95% CI)	P Value
Age group, y		
15-19	1(Reference)	
20-24	0.52 (0.13-2.19)	0.375
25-29	0.52 (0.15-1.78)	0.295
30-34	0.13 (0.04-0.47)	0.002
35-40	0.19 (0.06-0.64)	0.007
40-44	0.33 (0.10-1.09)	0.069
45-49	0.43 (0.13-1.42)	0.167
50-54	0.92 (0.27-3.08)	0.889
Education		
Primary school unfinished	1(Reference)	
Primary school finished	0.55 (0.37-0.81)	0.003
Secondary school unfinished	0.99 (0.79-1.24)	0.906
Secondary school or above	1.45 (1.10-1.92)	0.009
Household wealth quintile		
Q1 (Poorest)	1(Reference)	
Q2	0.46 (0.33-0.63)	<0.001
Q3	0.44 (0.31-0.61)	<0.001
Q4	0.29 (0.21-0.42)	<0.001
Q5 (Richest)	0.71 (0.51-1.01)	0.054
Currently married	3.07 (2.08-4.53)	<0.001
Female	2.71 (2.20-3.34)	<0.001
Urban	1.48 (1.21-1.81)	<0.001

Abbreviations: RR = Relative Risk; CI = Confidence Interval; Q = Quintile

[1] These regressions were run among those with co-morbid diabetes and hypertension, whereby aware, treated, and controlled refers to being aware, treated, and controlled for both conditions.

[2] The regressions included all variables listed in the table and an additional binary indicator for each of 640 districts (district-level fixed effects) as independent variables. All standard errors were adjusted for clustering at the level of a primary sampling unit.

eTable12. Individual-level correlates of reaching each cascade step among those with co-morbid diabetes and hypertension, women only[1,2]

Aware

	RR (95% CI)	P Value
Age group, y		
15-19	1(Reference)	
20-24	1.49 (0.77-2.88)	0.238
25-29	1.37 (0.72-2.64)	0.339
30-34	1.24 (0.65-2.36)	0.517
35-40	1.33 (0.70-2.53)	0.377
40-44	1.40 (0.74-2.65)	0.295
45-49	1.66 (0.88-3.14)	0.115
Education		
Primary school unfinished	1(Reference)	
Primary school finished	1.11 (0.97-1.26)	0.121
Secondary school unfinished	1.14 (1.04-1.25)	0.005
Secondary school or above	1.19 (1.06-1.35)	0.004
Household wealth quintile		
Q1 (Poorest)	1(Reference)	
Q2	1.08 (0.92-1.28)	0.347
Q3	1.18 (1.01-1.39)	0.043
Q4	1.34 (1.14-1.57)	<0.001
Q5 (Richest)	1.34 (1.13-1.58)	0.001
Currently married	1.08 (0.96-1.22)	0.182
Urban	1.24 (1.13-1.35)	<0.001

Treated

	RR (95% CI)	P Value
Age group, y		
15-19	1(Reference)	
20-24	0.41 (0.12-1.36)	0.146
25-29	0.63 (0.24-1.70)	0.366
30-34	0.56 (0.21-1.45)	0.23
35-40	0.80 (0.32-2.05)	0.647
40-44	1.08 (0.43-2.71)	0.876
45-49	1.45 (0.58-3.65)	0.431
Education		
Primary school unfinished	1(Reference)	
Primary school finished	1.11 (0.91-1.37)	0.311
Secondary school unfinished	1.06 (0.92-1.22)	0.446
Secondary school or above	1.10 (0.91-1.33)	0.319
Household wealth quintile		
Q1 (Poorest)	1(Reference)	
Q2	1.45 (1.08-1.95)	0.014
Q3	1.56 (1.17-2.09)	0.002
Q4	1.82 (1.37-2.41)	<0.001
Q5 (Richest)	2.03 (1.52-2.72)	<0.001
Currently married	1.14 (0.95-1.36)	0.17
Urban	1.60 (1.39-1.84)	<0.001

Controlled

	RR (95% CI)	P Value
Age group, y		
15-19	1(Reference)	
20-24	0.43 (0.12-1.57)	0.201
25-29	0.30 (0.09-0.97)	0.045
30-34	0.13 (0.04-0.43)	0.001
35-40	0.21 (0.07-0.64)	0.006
40-44	0.25 (0.09-0.72)	0.01
45-49	0.35 (0.12-0.99)	0.049
Education		
Primary school unfinished	1(Reference)	
Primary school finished	1.20 (0.76-1.89)	0.443
Secondary school unfinished	0.87 (0.62-1.23)	0.433
Secondary school or above	1.22 (0.78-1.91)	0.389
Household wealth quintile		
Q1 (Poorest)	1(Reference)	
Q2	0.98 (0.58-1.67)	0.953
Q3	0.98 (0.59-1.62)	0.932
Q4	1.40 (0.86-2.28)	0.181
Q5 (Richest)	1.29 (0.76-2.21)	0.346
Currently married	1.31 (0.86-2.01)	0.213
Urban	1.67 (1.22-2.29)	0.001

Abbreviations: RR = Relative Risk; CI = Confidence Interval; Q = Quintile

[1] These regressions were run among those with co-morbid diabetes and hypertension, whereby aware, treated, and controlled refers to being aware, treated, and controlled for both conditions.

[2] The regressions included all variables listed in the table and an additional binary indicator for each of 640 districts (district-level fixed effects) as independent variables. All standard errors were adjusted for clustering at the level of a primary sampling unit.

eTable13. Individual-level correlates of reaching each cascade step among those with co-morbid diabetes and hypertension, men only[1,2]

Aware

	**RR (95% CI)**	**P Value**
Age group, y		
15-19	1(Reference)	
20-24	0.43 (0.09-2.02)	0.283
25-29	0.71 (0.21-2.35)	0.575
30-34	0.47 (0.14-1.53)	0.209
35-40	0.75 (0.25-2.28)	0.609
40-44	1.04 (0.36-3.01)	0.948
45-49	1.32 (0.45-3.87)	0.618
50-54	1.47 (0.50-4.33)	0.48
Education		
Primary school unfinished	1(Reference)	
Primary school finished	1.14 (0.66-1.96)	0.647
Secondary school unfinished	1.24 (0.91-1.67)	0.17
Secondary school or above	1.28 (0.91-1.81)	0.153
Household wealth quintile		
Q1 (Poorest)	1(Reference)	
Q2	1.22 (0.75-2.00)	0.424
Q3	1.25 (0.76-2.04)	0.378
Q4	1.21 (0.73-2.00)	0.466
Q5 (Richest)	1.63 (0.97-2.74)	0.064
Currently married	0.75 (0.47-1.19)	0.222
Urban	1.31 (0.99-1.72)	0.055

Abbreviations: RR = Relative Risk; CI = Confidence Interval; Q = Quintile

[1] These regressions were run among those with co-morbid diabetes and hypertension, whereby aware, treated, and controlled refers to being aware, treated, and controlled for both conditions.

[2] The regressions included all variables listed in the table and an additional binary indicator for each of 640 districts (district-level fixed effects) as independent variables. All standard errors were adjusted for clustering at the level of a primary sampling unit.

Treated[1]

	**RR (95% CI)**	**P Value**
Age group, y		
15-24	1(Reference)	
25-34	1.08 (0.17-7.03)	0.934
35-44	2.19 (0.31-15.56)	0.432
45-54	4.25 (0.62-29.38)	0.142
Education		
Primary school unfinished	1(Reference)	
Primary school finished	2.01 (0.80-5.01)	0.136
Secondary school unfinished	1.38 (0.78-2.44)	0.273
Secondary school or above	1.62 (0.85-3.08)	0.141
Household wealth quintile		
Q1 (Poorest)	1(Reference)	
Q2	0.87 (0.40-1.88)	0.724
Q3	0.61 (0.28-1.35)	0.224
Q4	0.79 (0.35-1.79)	0.573
Q5 (Richest)	1.19 (0.52-2.72)	0.682
Currently married	0.94 (0.43-2.08)	0.885
Urban	1.74 (1.11-2.72)	0.016

[1] Age groups were larger in this regression (10 years) due to the fact that the age groups used in previous regressions (5 years) were too small for this subgroup, resulting in empty groups.

Controlled[1]

	RR (95% CI)	P Value
Age group, y		
15-28	1(Reference)	
29-41	0.19 (0.04-0.89)	0.035
42-54	0.60 (0.17-2.14)	0.43
Education		
Primary school unfinished	1(Reference)	
Primary school finished	0.92 (0.07-12.75)	0.95
Secondary school unfinished	2.20 (0.41-11.68)	0.355
Secondary school or above	2.65 (0.45-15.75)	0.284
Household wealth quintile		
Q1 (Poorest)	1(Reference)	
Q2	0.20 (0.05-0.76)	0.019
Q3	0.17 (0.03-0.79)	0.024
Q4	0.09 (0.02-0.56)	0.009
Q5 (Richest)	0.32 (0.07-1.49)	0.148
Currently married	1.88 (0.29-12.18)	0.508
Urban	1.98 (0.84-4.65)	0.117

[1] Age groups were larger in this regression (10 years) due to the fact that the age groups used in previous regressions (5 years) were too small for this subgroup, resulting in empty groups.

eTable14. Diabetes cascade estimates with comorbidities

Care indicator	Comorbidity	Estimate (in %)	Low CI (in %)	High CI (in %)
Aware	No other CVD risk factor	55.83	53.04	58.58
Treated	No other CVD risk factor	39.92	37.35	42.55
Controlled	No other CVD riskfactor	27.86	25.46	30.41
Aware	One other CVD risk factor	52.85	50.30	55.38
Treated	One other CVD risk factor	41.56	39.04	44.12
Controlled	One other CVD risk factor	24.22	21.86	26.75
Aware	Two other CVD risk factors	50.50	46.84	54.15
Treated	Two other CVD risk factors	43.87	40.21	47.59
Controlled	Two other CVD risk factors	21.59	18.87	24.58
Aware	Three other CVD risk factors	49.07	40.72	57.46
Treated	Three other CVD risk factors	40.74	32.67	49.35
Controlled	Three other CVD risk factors	18.56	12.23	27.14
Aware	No Asthma/Anemia	49.06	47.14	50.98
Treated	No Asthma/Anemia	40.02	38.17	41.89
Controlled	No Asthma/Anemia	22.20	20.67	23.81
Aware	Asthma or Anemia	66.13	62.52	69.57
Treated	Asthma or Anemia	46.24	42.02	50.51
Controlled	Asthma or Anemia	31.08	27.37	35.05
Aware	Asthma and Anemia	87.94	79.68	93.13
Treated	Asthma and Anemia	68.05	56.63	77.65
Controlled	Asthma and Anemia	51.48	38.79	63.99

eTable15. Hypertension cascade estimates with comorbidities

Care indicator	Comorbidity	Estimate (in %)	Low CI (in %)	High CI (in %)
Screened	No other CVD risk factor	78.08	77.18	78.96
Aware	No other CVD risk factor	48.69	47.44	49.94
Treated	No other CVD risk factor	12.51	12.00	13.04
Controlled	No other CVD risk factor	8.42	8.00	8.85
Screened	One other CVD risk factor	73.18	72.07	74.26
Aware	One other CVD risk factor	39.12	37.79	40.46
Treated	One other CVD risk factor	13.29	12.55	14.06
Controlled	One other CVD risk factor	6.95	6.39	7.55
Screened	Two other CVD risk factors	78.95	76.65	81.08
Aware	Two other CVD risk factors	43.44	40.77	46.15
Treated	Two other CVD risk factors	23.56	21.57	25.66
Controlled	Two other CVD risk factors	9.93	8.57	11.49
Screened	Three other CVD risk factors	84.75	78.25	89.56
Aware	Three other CVD risk factors	48.87	39.18	58.66
Treated	Three other CVD risk factors	25.15	18.09	33.83
Controlled	Three other CVD risk factors	8.54	4.61	15.27
Screened	No Asthma/Anemia	74.90	74.07	75.72
Aware	No Asthma/Anemia	41.94	40.80	43.10
Treated	No Asthma/Anemia	13.09	12.59	13.61
Controlled	No Asthma/Anemia	7.36	6.97	7.76
Screened	Asthma or Anemia	82.89	81.80	83.93
Aware	Asthma or Anemia	56.79	55.26	58.30
Treated	Asthma or Anemia	17.59	16.68	18.55
Controlled	Asthma or Anemia	10.99	10.32	11.70
Screened	Asthma and Anemia	89.33	84.09	92.99
Aware	Asthma and Anemia	64.93	57.92	71.36
Treated	Asthma and Anemia	27.60	20.93	35.44
Controlled	Asthma and Anemia	13.14	9.46	17.97

eTable16. Combined cascade estimates with comorbidities

Care indicator	Comorbidity	Estimate (in %)	Low CI (in %)	High CI (in %)
Aware	No other CVD risk factor	28.88	26.14	31.79
Treated	No other CVD risk factor	13.68	11.61	16.06
Controlled	No other CVD risk factor	3.29	2.32	4.65
Aware	One other CVD risk factor	29.45	26.40	32.70
Treated	One other CVD risk factor	18.21	15.72	21.00
Controlled	One other CVD risk factor	4.45	3.03	6.50
Aware	Two other CVD risk factors	24.21	16.93	33.36
Treated	Two other CVD risk factors	13.90	8.21	22.56
Controlled	Two other CVD risk factors	1.41	0.39	4.95
Aware	No Asthma/Anemia	26.30	24.05	28.67
Treated	No Asthma/Anemia	15.10	13.22	17.19
Controlled	No Asthma/Anemia	3.40	2.39	4.82
Aware	Asthma or Anemia	38.36	33.53	43.43
Treated	Asthma or Anemia	19.60	16.34	23.34
Controlled	Asthma or Anemia	5.21	3.69	7.30
Aware	Asthma and Anemia	73.08	53.40	86.54
Treated	Asthma and Anemia	35.92	16.25	61.82
Controlled	Asthma and Anemia	4.97	1.91	12.30

eTable17. 'Treated' for tobacco consumption with comorbidities

State	Estimate (in %)	Low CI (in %)	Upper CI (in %)	State
Treated	No other CVD risk factor	50.87	48.48	53.26
Treated	One other CVD risk factor	55.99	52.85	59.09
Treated	Two other CVD risk factors	58.94	51.41	66.07
Treated	Three other CVD risk factors	47.76	27.76	68.50
Treated	No Asthma/Anemia	51.94	49.70	54.18
Treated	Asthma or Anemia	55.27	51.69	58.79
Treated	Asthma and Anemia	72.18	54.67	84.81

eTable 18. Univariable regressions: The association of having multiple cardiovascular disease risk factors with the probability of reaching each cascade step[1]

	BP ever measured		Aware of hypertension		Hypertension treated		Hypertension controlled	
Has hypertension and …	*RR (95% CI)*	*P*	*RR (95% CI)*	*P*	*RR (95% CI)*	*P*	*RR (95% CI)*	*P*
diabetes	1.34 (1.27-1.43)	< 0.001	1.10 (1.08-1.12)	< 0.001	1.84 (1.77-1.91)	< 0.001	1.34 (1.27-1.43)	< 0.001
obesity	1.15 (1.13-1.17)	< 0.001	1.07 (1.05-1.08)	< 0.001	1.56 (1.51-1.60)	< 0.001	1.16 (1.12-1.21)	< 0.001
smokes	0.90 (0.88-0.92)	< 0.001	0.79 (0.77-0.80)	< 0.001	0.81 (0.78-0.84)	< 0.001	0.72 (0.69-0.76)	< 0.001
	-		**Aware of diabetes**		**Diabetes treated**		**Diabetes controlled**	
Has diabetes and …	-	-	*RR (95% CI)*	*P*	*RR (95% CI)*	*P*	*RR (95% CI)*	*P*
hypertension	-	-	0.97 (0.95-1.00)	0.052	1.08 (1.05-1.12)	< 0.001	0.82 (0.78-0.86)	< 0.001
obesity	-	-	0.97 (0.94-1.00)	0.060	1.07 (1.03 -1.11)	< 0.001	0.85 (0.80-0.90)	< 0.001
smokes	-	-	0.93 (0.90-0.97)	< 0.001	0.89 (0.85-0.93)	< 0.001	0.89 (0.84-0.96)	0.001
	-		**Aware of both**		**Both treated**		**Both controlled**	
Has diabetes, hypertension, and …	-	-	*RR (95% CI)*	*P*	*RR (95% CI)*	*P*	*RR (95% CI)*	*P*
obesity	-	-	1.12 (1.05-1.20)	0.001	2.34 (2.10-2.64)	< 0.001	2.04 (1.62-2.55)	< 0.001
smokes	-	-	0.80 (0.73-0.88)	< 0.001	0.77 (0.66-0.90)	0.001	0.74 (0.53-1.02)	0.070

Abbreviations: RR = Relative Risk; CI = Confidence Interval

[1] The univariable regressions were run separately for each care cascade step and contained one of the listed indicators in the table (hypertension, diabetes, smoking) and a binary indicator for each of 640 districts (district-level fixed effects) as independent variables. Standard errors were adjusted for clustering at the level of a primary sampling unit.

eTable 19. The association of having multiple cardiovascular disease risk factors with the probability of reaching each cascade step using sampling weights[1]

	**BP ever measured**		**Aware of hypertension**		**Hypertension treated**		**Hypertension controlled**	
Has hypertension and …	*RR (95% CI)*	*P*	*RR (95% CI)*	*P*	*RR (95% CI)*	*P*	*RR (95% CI)*	*P*
diabetes	1.04 (1.03-1.06)	<0.001	1.11 (1.09-1.13)	<0.001	1.58 (1.54-1.63)	<0.001	1.46 (1.40-1.53)	<0.001
obesity	1.02 (1.01-1.04)	<0.001	1.02 (1.01-1.04)	0.006	1.42 (1.39-1.46)	<0.001	1.16 (1.12-1.20)	<0.001
smokes	0.98 (0.97-0.99)	0.001	0.99 (0.98-1.01)	0.32	0.91 (0.89-0.94)	<0.001	0.85 (0.82-0.89)	<0.001
	-		**Aware of diabetes**		**Diabetes treated**		**Diabetes controlled**	
Has diabetes and …	-	-	*RR (95% CI)*	*P*	*RR (95% CI)*	*P*	*RR (95% CI)*	*P*
hypertension	-	-	0.98 (0.95-1.00)	0.108	1.07 (1.04-1.10)	<0.001	0.91 (0.88-0.95)	<0.001
obesity	-	-	0.94 (0.92-0.97)	<0.001	0.97 (0.94-1.00)	0.084	0.83 (0.80-0.87)	<0.001
smokes	-	-	0.99 (0.95-1.02)	0.397	0.97 (0.94-1.01)	0.106	1.08 (1.03-1.13)	0.003
	-		**Aware of both**		**Both treated**		**Both controlled**	
Has diabetes, hypertension, and …	-	-	*RR (95% CI)*	*P*	*RR (95% CI)*	*P*	*RR (95% CI)*	*P*
obesity	-	-	1.08 (1.02-1.14)	0.409	1.48 (1.37-1.60)	<0.001	1.49 (1.26-1.76)	<0.001
smokes	-	-	0.98 (0.91-1.05)	0.893	0.79 (0.71-0.87)	<0.001	0.73 (0.58-0.92)	0.006

Abbreviations: RR = Relative Risk; CI = Confidence Interval

[1] The regressions were run separately for each care cascade step and – in addition to the cardiovascular disease risk factor shown in the table – contained age group, household wealth quintile, education, currently married, urban vs. rural location, sex, and a binary indicator for each of 640 districts (district-level fixed effects) as independent variables. Standard errors were adjusted for clustering at the level of a primary sampling unit and sampling weights were used that also accounted for the lower probability of sampling men.

eTable 20. The association of having multiple cardiovascular disease risk factors with the probability of reaching each cascade step, men only[1]

	**BP ever measured**		**Aware of hypertension**		**Hypertension treated**		**Hypertension controlled**	
Has hypertension and …	*RR (95% CI)*	*P*	*RR (95% CI)*	*P*	*RR (95% CI)*	*P*	*RR (95% CI)*	*P*
diabetes	1.06 (1.04-1.09)	<0.001	1.18 (1.11-1.26)	<0.001	1.59 (1.43-1.76)	<0.001	1.57 (1.33-1.85)	<0.001
obesity	1.03 (1.01-1.05)	0.007	1.06 (1.01-1.11)	0.025	1.37 (1.25-1.50)	<0.001	1.12 (0.97-1.29)	0.114
smokes	0.98 (0.96-1.00)	0.018	0.99 (0.95-1.03)	0.634	0.93 (0.85-1.02)	0.109	0.89 (0.78-1.01)	0.06
	**-**		**Aware of diabetes**		**Diabetes treated**		**Diabetes controlled**	
Has diabetes and …	-	-	*RR (95% CI)*	*P*	*RR (95% CI)*	*P*	*RR (95% CI)*	*P*
hypertension	-	-	0.93 (0.86-0.99)	0.029	0.98 (0.89-1.07)	0.586	0.82 (0.71-0.94)	0.004
obesity	-	-	0.93 (0.85-1.01)	0.071	0.95 (0.85-1.05)	0.324	0.81 (0.68-0.97)	0.024
smokes	-	-	0.94 (0.88-1.01)	0.104	0.92 (0.84-1.00)	0.059	0.94 (0.83-1.07)	0.354
	**-**		**Aware of both**		**Both treated**		**Both controlled**	
Has diabetes, hypertension, and …	-	-	*RR (95% CI)*	*P*	*RR (95% CI)*	*P*	*RR (95% CI)*	*P*
obesity	-	-	1.06 (0.84-1.34)	0.609	1.30 (0.89-1.88)	0.17	1.72 (0.80-3.67)	0.163
smokes	-	-	1.00 (0.80-1.25)	0.998	0.90 (0.64-1.26)	0.532	1.00 (0.42-2.36)	0.999

Abbreviations: RR = Relative Risk; CI = Confidence Interval

[1] The regressions were run separately for each care cascade step and – in addition to the cardiovascular disease risk factor shown in the table – contained age group, household wealth quintile, education, currently married, urban vs. rural location, sex, and a binary indicator for each of 640 districts (district-level fixed effects) as independent variables. Standard errors were adjusted for clustering at the level of a primary sampling unit. The analysis was only performed in the male sample.

eTable 21. The association of having multiple cardiovascular disease risk factors with the probability of reaching each cascade step, women only[1]

	**BP ever measured**		**Aware of hypertension**		**Hypertension treated**		**Hypertension controlled**	
Has hypertension and …	*RR (95% CI)*	*P*	*RR (95% CI)*	*P*	*RR (95% CI)*	*P*	*RR (95% CI)*	*P*
diabetes	1.04 (1.03-1.05)	<0.001	1.11 (1.08-1.13)	<0.001	1.50 (1.44-1.56)	<0.001	1.29 (1.21-1.37)	<0.001
obesity	1.02 (1.01-1.03)	<0.001	1.01 (0.99-1.02)	0.421	1.29 (1.25-1.33)	<0.001	1.07 (1.02-1.12)	0.003
smokes	0.99 (0.98-1.00)	0.15	0.99 (0.96-1.01)	0.193	1.00 (0.95-1.05)	0.963	1.01 (0.95-1.08)	0.739
	-		**Aware of diabetes**		**Diabetes treated**		**Diabetes controlled**	
Has diabetes and …	-	-	*RR (95% CI)*	*P*	*RR (95% CI)*	*P*	*RR (95% CI)*	*P*
hypertension	-	-	0.96 (0.93-0.99)	0.008	1.00 (0.96-1.04)	0.981	0.83 (0.78-0.88)	<0.001
obesity	-	-	0.91 (0.88-0.94)	<0.001	0.93 (0.90-0.97)	<0.001	0.82 (0.77-0.87)	<0.001
smokes	-	-	1.07 (1.02-1.13)	0.007	1.06 (1.00-1.13)	0.069	1.10 (1.00-1.20)	0.045
	-		**Aware of both**		**Both treated**		**Both controlled**	
Has diabetes, hypertension, and …	-	-	*RR (95% CI)*	*P*	*RR (95% CI)*	*P*	*RR (95% CI)*	*P*
obesity	-	-	1.00 (0.93-1.08)	0.965	1.30 (1.16-1.45)	<0.001	1.20 (0.92-1.57)	0.174
smokes	-	-	1.03 (0.90-1.17)	0.658	1.03 (0.83-1.27)	0.794	0.99 (0.60-1.64)	0.969

Abbreviations: RR = Relative Risk; CI = Confidence Interval

[1] The regressions were run separately for each care cascade step and – in addition to the cardiovascular disease risk factor shown in the table – contained age group, household wealth quintile, education, currently married, urban vs. rural location, sex, and a binary indicator for each of 640 districts (district-level fixed effects) as independent variables. Standard errors were adjusted for clustering at the level of a primary sampling unit. The analysis was only performed in the female sample.

eFigure 1. Combined care cascade: states' gross domestic product (GDP) per capita and each care cascade step[1,2,3,4,5,6,7]

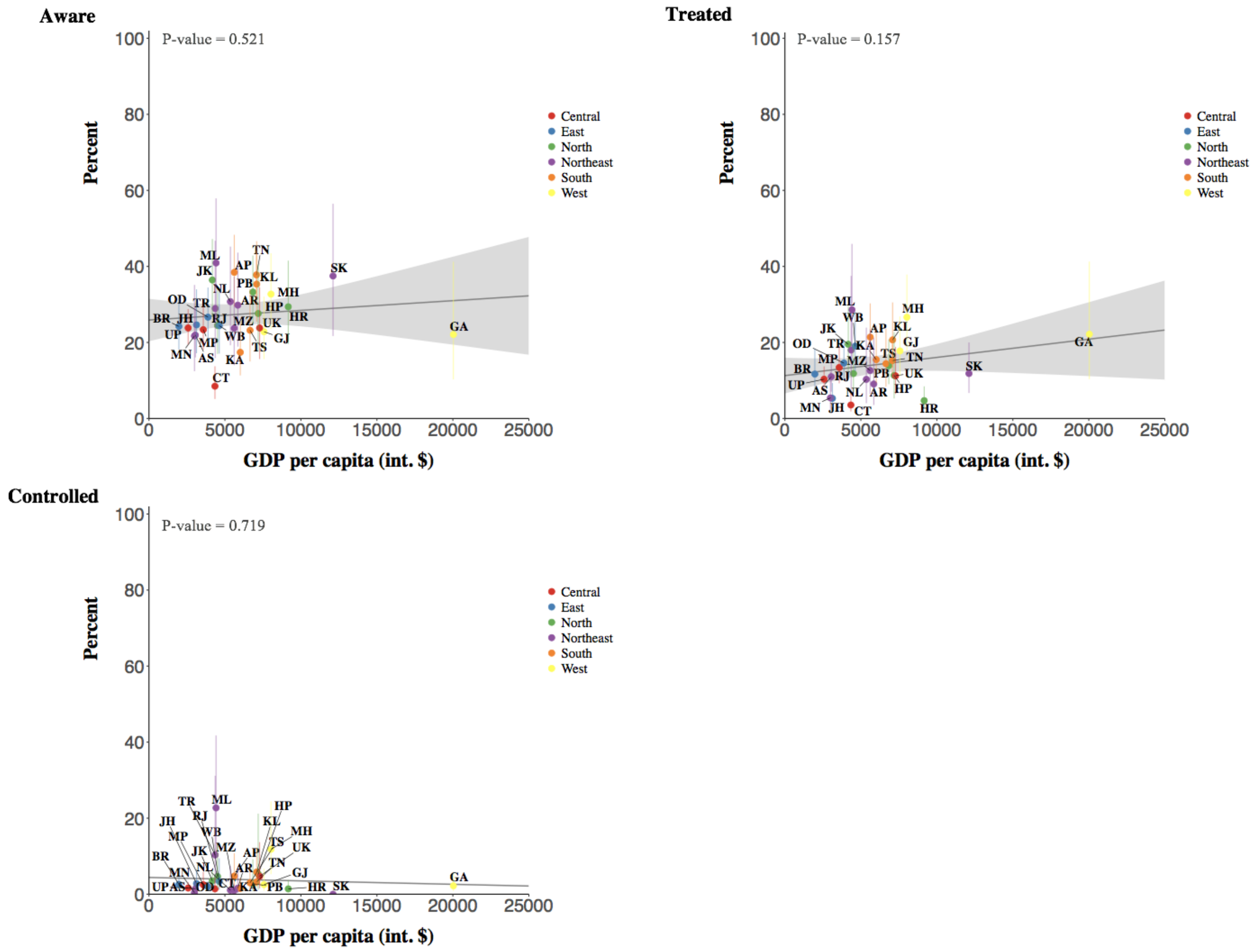

GDP=Gross Domestic Product; int. = international.

[1]State population estimates from the 2011 Census of India and GDP data (for 2013/2014) from the Central Statistics Office, Ministry of Statistics and Programme Implementation, Government of India were used.(3, 4) Indian Rupees were converted to 2015 international dollars in accordance with the purchasing power parity conversion factors published by the World Bank.(5)

[2] The grey ribbon represents the point-wise 95% prediction interval, whereas the vertical bars depict 95% confidence intervals.

[3]P-values refer to the regression coefficient of the ordinary least squares regression line (with each state having the same weight) shown in the figure.

[4]States were divided into regions in accordance with their allocation to Zonal Councils by the Government of India.(6)

[5]Union Territories were excluded from this figure, since GDP per capita information was not available for three of seven Union Territories.

[6]AP indicates Andhra Pradesh; AR, Arunachal Pradesh; AS, Assam; BR, Bihar; CT, Chhattisgarh; GA, Goa; GJ, Gujarat; HR, Haryana; HP, Himachal Pradesh; JH, Jharkhand; JK, Jammu and Kashmir; KA, Karnataka; KL,

Kerala; MP, Madhya Pradesh; MH, Maharashtra; MN, Manipur; ML, Meghalaya; MZ, Mizoram; NL, Nagaland; OD, Odisha (Orissa); PB, Punjab; RJ, Rajasthan; SK, Sikkim; TN, Tamil Nadu; TS, Telangana State; TR, Tripura; UP, Uttar Pradesh; UK, Uttarakhand (Uttaranchal); WB, West Bengal
[7]Estimates can be found in **eTable4-6**.

eFigure 2. 'Treated' for smoking tobacco with additional CVD-risk factors or non-CVD morbidites[1,2]

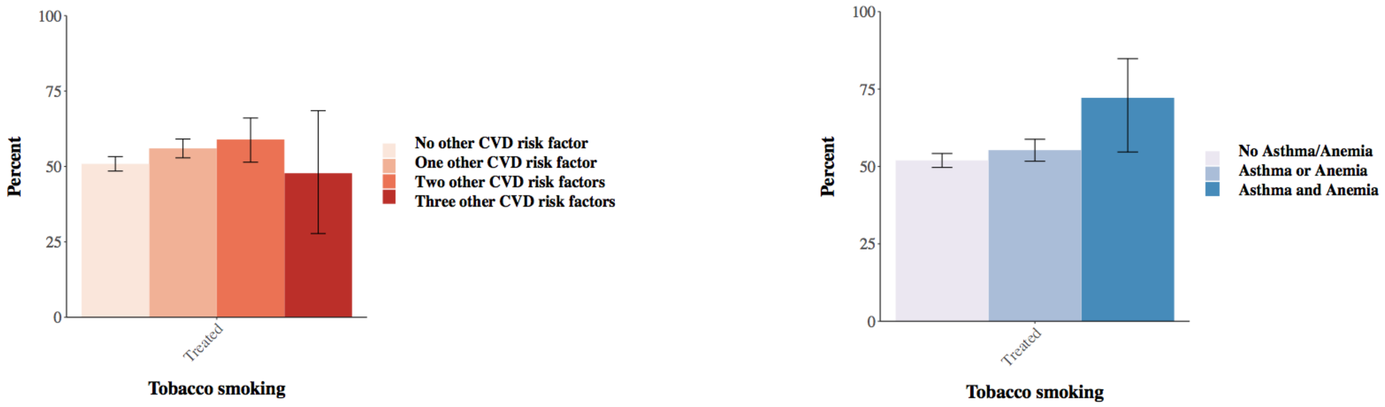

[1]Estimates can be found in **eTable17.**
[2]All estimates are weighted using sampling weights that also accounted for the lower probability of sampling men.

References

1. The DHS Program, Wealth Index construction, https://dhsprogram.com/topics/wealth-index/Wealth-Index-Construction.cfm.
2. The DHS Program, Steps to constructing the new DHS Wealth Index, https://dhsprogram.com/programming/wealth%20index/Steps_to_constructing_the_new_DHS_Wealth_Index.pdf.
3. Government of India MoSaPI. State Domestic Product and other aggregates with current prices, 2004-05 series. http://wwwmospigovin/data. 2013-2014.
4. Government of India MoHaFW. State/ UT-wise Rural and Urban Population as Per Census during 2001 and 2011. https://datagovin/resources/state-ut-wise-rural-and-urban-population-census-during-2001-and-2011. 2011.
5. Group WB. International Comparison Program database. https://dataworldbankorg/indicator/PANUSPPP. 2018 (accessed online 30.07.2018).
6. Ministry of Home Affairs. Meetings of Zonal Councils New Delhi: Government of India; 2017 [Available from: http://mha.nic.in/zonal_council.
